# Krupple-Like Factor 5 is a Potential Therapeutic Target and Prognostic Marker in Epithelial Ovarian Cancer

**DOI:** 10.3389/fphar.2020.598880

**Published:** 2020-12-03

**Authors:** Abdul K. Siraj, Poyil Pratheeshkumar, Sasidharan Padmaja Divya, Sandeep Kumar Parvathareddy, Khadija A. Alobaisi, Saravanan Thangavel, Sarah Siraj, Ismail A. Al-Badawi, Fouad Al-Dayel, Khawla S. Al-Kuraya

**Affiliations:** ^1^ Human Cancer Genomic Research, King Faisal Specialist Hospital and Research Center, Riyadh, Saudi Arabia; ^2^ Department of Obstetrics and Gynecology, King Faisal Specialist Hospital and Research Centre, Riyadh, Saudi Arabia; ^3^ Department of Pathology, King Faisal Specialist Hospital and Research Centre, Riyadh, Saudi Arabia

**Keywords:** epithelial ovarian cancer, Krupple-like factor 5, pSTAT3, epithelial–mesenchymal transition, apoptosis

## Abstract

Epithelial ovarian cancer (EOC) is the most lethal gynecological malignancy. Despite current therapeutic and surgical options, advanced EOC shows poor prognosis. Identifying novel molecular therapeutic targets is highly needed in the management of EOC. Krupple-like factor 5 (KLF5), a zinc-finger transcriptional factor, is highly expressed in a variety of cancer types. However, its role and expression in EOC is not fully illustrated. Immunohistochemical analysis was performed to assess KLF5 protein expression in 425 primary EOC samples using tissue microarray. We also addressed the function of KLF5 in EOC and its interaction with signal transducer and activator of transcription 3 (STAT3) signaling pathway. We found that KLF5 overexpressed in 53% (229/425) of EOC samples, and is associated with aggressive markers. Forced expression of KLF5 enhanced cell growth in low expressing EOC cell line, MDAH2774. Conversely, knockdown of KLF5 reduced cell growth, migration, invasion and progression of epithelial to mesenchymal transition in KLF5 expressing cell lines, OVISE and OVSAHO. Importantly, silencing of KLF5 decreased the self-renewal ability of spheroids generated from OVISE and OVSAHO cell lines. In addition, downregulation of KLF5 potentiated the effect of cisplatin to induce apoptosis in these cell lines. These data reveals the pro-tumorigenic role of KLF5 in EOC and uncover its role in activation of STAT3 signaling pathway, suggesting the importance of KLF5 as a potential therapeutic target for EOC therapy.

## Introduction

The current standard therapy for EOC is cytoreductive surgery followed by platinum-based chemotherapy, with reported response rate of over 70%. However, resistance to platinum-based chemotherapy is considered one of the most important causes of failure of EOC therapy and decrease in the overall 5-years survival rate of EOC (Ushijima, 2010). Therefore, there is an urgent need to identify new, more effective treatment strategies for patients with this lethal disease and identify new targets that also could potentially improve platinum-based therapy.

Krupple-like factor 5 (KLF5) is a zinc-Finger transcription factor involved in many cellular functions such as differentiation, proliferation, migration, apoptosis and regulation of cancer stemness (Mori et al., 2009; Maehara et al., 2015; Hayashi et al., 2016; Ma et al., 2017; Li et al., 2018; Ma et al., 2018; Li et al., 2019). KLF5 expression and activity are altered in many cancer types including breast (Takagi et al., 2012), cervical (Ma et al., 2017), colorectal (Ghaleb and Yang, 2008), lung (Li et al., 2014), pancreatic (Li et al., 2019) and thyroid cancer (Ma et al., 2018). Clinically KLF5 has been found to play an oncogenic role where it is associated with tumor progression, aggressive clinical behavior and poor survival (Tong et al., 2006; Takagi et al., 2012; Dong et al., 2013; Li et al., 2014; Ma et al., 2017; Li et al., 2019).

The oncogene, signal transducer and activator of transcription 3 (STAT3), is found constitutively activated in many human malignancies (Levy and Inghirami, 2006; Zhang and Lai, 2014; Guanizo et al., 2018). Activated STAT3 regulates a variety of tumor cell processes, such as tumor cell growth, survival, invasion, cancer stemness, and chemoresistance, and has been shown to contribute to disease aggressiveness in ovarian cancer (Saini et al., 2017; Liang et al., 2020; Wu et al., 2020). Constitutively active STAT3 up-regulates the survival factors, Bcl-2 and Bcl-xL, whilst inhibiting apoptosis via induction of AKT (Siddiquee and Turkson, 2008). A recent study showed KLF5 disruption can reduce STAT3 activation, and cause tumor regression *in vivo* (He et al., 2018).

Previous study has shown that KLF5 is highly expressed in ovarian cancer cell line SKOV3 sphere cells and its silencing could sensitized the sphere cells to apoptosis induced by chemotherapeutic drug (Dong et al., 2013). Therefore, in this study we sought to explore in details the KLF5 expression, cellular function and regulation both clinically and functionally. Upon investigating the clinical role of KLF5 in more than 400 EOC, we found strong correlation with STAT3 activation in a subset of EOC cases. This prompt us to further study the function of KLF5 in EOC cell lines and its interaction with STAT3 signaling pathway. This study provides strong evidence of the oncogenic role of KLF5 in EOC, its inhibition downregulate STAT3 and potentiate the apoptotic effect induced by chemotherapeutic drug (cisplatin). Overall, this study suggests the importance of KLF5 as a potential therapeutic target for EOC therapy.

## Materials and Methods

### Sample Selection

Archival samples from 425 EOC patients diagnosed between 1989 and 2017 at King Faisal Specialist Hospital and Research Center (Riyadh, Saudi Arabia) were included in the study. Detailed clinicopathological data were noted from case records and have been summarized in Table 1. All samples were obtained from patients with approval from Institutional Review Board of the hospital. For the study, since only archived paraffin tissue blocks were used, a waiver of consent was obtained from Research Advisory Council (RAC) under project RAC# 2140033.

**TABLE 1:** Clinicopathological variables for the patient cohort (*n* = 425).

	*n* (%)
Age	
Median	50.0
Range (IQR)^a^	41.0–62.0
Histopathology	
Serous	307 (72.2)
Mucinous	62 (14.7)
Endometrioid	38 (8.9)
Clear cell	9 (2.1)
Undifferentiated	9 (2.1)
Histological grade	
Grade 1	89 (20.9)
Grade 2	147 (34.6)
Grade 3	176 (41.4)
Unknown	13 (3.1)
pT	
T1	89 (20.9)
T2	38 (8.9)
T3	293 (68.9)
Unknown	5 (1.2)
pN	
N0	386 (90.8)
N1	34 (8.0)
Unknown	5 (1.2)
pM	
M0	345 (81.2)
M1	75 (17.6)
Unknown	5 (1.2)
Stage	
I	83 (19.5)
II	24 (5.6)
III	241 (56.7)
IV	66 (15.5)
Unknown	11 (2.6)

^a^Inter quartile range

### Tissue Microarray Construction and Immunohistochemistry Staining

#### Tissue Microarray

All samples were analyzed in a tissue microarray (TMA) format. TMA construction was performed as described earlier (Siraj et al., 2007). Briefly, tissue cylinders with a diameter of 0.6 mm were punched from representative tumor regions of each donor tissue block and brought into recipient paraffin block using a modified semiautomatic robotic precision instrument (Beecher Instruments, Woodland, WI). Two cores of EOC were arrayed from each case.

### Immunohistochemistry (IHC) Staining and Evaluation

Standard protocol was followed for IHC staining. For antigen retrieval, Dako (Dako Denmark A/S, Glostrup, Denmark) Target Retrieval Solution pH 9.0 (Catalog number S2368) was used, and the slides were placed in Pascal pressure cooker at 120 °C for 10 min. The slides were incubated with primary antibody against KLF5 (HPA040398, 1:100 dilution, pH 6.0, Sigma Aldrich, St. Louis, Missouri, USA) and pSTAT-3 Tyr-705 (1:100 dilution, pH 9.0, Cell Signaling Technology, Danvers, Massachusetts, USA). The Dako Envision Plus System kit was used as the secondary detection system with 3, 30-diaminobenzidine as chromogen. All slides were counterstained with hematoxylin, dehydrated, cleared and mounted. Negative controls included omission of the primary antibody. Normal tissues of different organ system were also included in the TMA to serve as control. Only fresh cut slides were stained simultaneously to minimize the influence of slide aging and maximize reproducibility of the experiment. The slides were independently examined by two pathologists. If there was a discrepancy in the individual scores, both pathologists carried out a re-evaluation until a consensus was reached.

KLF5 and pSTAT-3 immunohistochemical expression were seen predominantly in the nuclear compartment and nuclear expression was quantified using the proportion score as described previously (Nakajima et al., 2011; Yoshikawa et al., 2018). Briefly, the proportion of positively stained tumor cells was calculated as a percentage for each core and the scores were averaged across two tissue cores from the same tumor to yield a single percent staining score representing each cancer patient. Cases showing expression level of more than 10% were classified as overexpression and those with ≤10% as low expression.

### Reagents and Antibodies

ML264 (KLF5 selective inhibitor) was purchased from MyBioSource, Inc (San Diego, CA). Cisplatin (P4394) and KLF5 antibody (HPA 040398) were obtained from Sigma (St. Louis, MO). Antibodies against pAKT (sc-7985), STAT3 (sc-482), caspase-3 (sc-56053), Cytochrome c (sc-13156), α-tubulin (sc-23948) and GAPDH (sc-25778) were purchased from Santa Cruz Biotechnology, Inc (Santa Cruz, CA). Antibodies against pSTAT3 (Tyr705) (9138), AKT (9272), pBad (9296), E-cadherin (3195), MMP-2 (13132), MMP-9 (2270), Bcl-2 (2876), Bcl-xl (2762), CIAP1 (4952), caspase-9 (9508), cleaved caspase-3 (9661), Cox IV (4844), PARP (9542), CD44 (3570), CD133 (64326), NANOG (4903) and OCT4 (2750) were purchased from Cell Signaling Technology (Danvers, MA). Antibodies against N-cadherin (ab98952), Vimentin (ab92547), and Twist (ab175430) were purchased from Abcam (Cambridge, England). XIAP antibody (610763) and *trans*-well invasion and migration kits were purchased from BD Biosciences (San Jose, CA).

### Cell Culture

EOC cell lines MDAH2774, SKOV3, OVCAR3, OVTOKO, OVISE and OVSAHO cells were purchased from (American Type Culture Collection, Manassas, VA). Following tests of these cell lines for immunological markers and cytogenetics, they were also fingerprinted and species was confirmed by IEF of AST, MDH and NP. The cell lines were cultured in RPMI 1640 supplemented with 10% (v/v) fetal bovine serum (ATCC), 100 units/mL penicillin, and 100 units/mL streptomycin (SIGMA) at 37°C in humidified atmosphere containing 5% CO_2_. All experiments were performed in RPMI 1640 (ATCC) containing 5% serum.

### Gene Silencing Using siRNA

KLF5 siRNA (SR300482), and scrambled control siRNA were purchased from OriGene (Rockville, MD). STAT3 siRNA (sc-29493), and scrambled control siRNA were purchased from Santa Cruz Biotechnology, Inc (Santa Cruz, CA). Cells (2 × 10^5^) were seeded in 6 well plate and transfected using Lipofectamine 2000 (Invitrogen, Carlsbad, CA) for 6 h following which the lipid and siRNA complex was removed and fresh growth medium was added. After 48 h of transfection, cells were used for immunoblotting.

### Plasmid and Transfection

Plasmid DNA encoding human *KLF5* (RC202438) and shRNA targeting human *KLF5* (TR311886) were purchased from Origene (Rockville, MD). The overexpression of KLF5 in EOC cells were performed using Lipofectamine™2000 (Invitrogen, Carlsbad, CA) according to the manufacturer's protocol. Briefly, EOC cells were seeded in 6-well culture plates; when approximately 50% confluent, cells were transfected with 4 μg plasmid. After 48 h of transfection, overexpression of KLF5 and knockdown of KLF5 protein production were confirmed by immunoblotting.

### MTT (3-(4,5-Dimethylthiazol-2-yl)-2,5-Diphenyltetrazolium Bromide) Assays

EOC cells were incubated at the concentration of 10^4^ cells per well in a 96 well format. Cells were then treated with various doses of ML264 for 48 h in a final volume of 0.2 ml. DMSO (0.01%) was used as vehicle control in all the ML264 treatments. Cell viability was measured by MTT assay. Six wells for each dosage including vehicle control were analyzed for each experiment.

### Cell Invasion and Migration Assays

Cell invasion and migration assay were performed as described previously (Bu et al., 2018). Briefly, cells after treatment with ML264 or siRNA knockdown for 48 h, cells were re-counted and equal number of cells were seeded into *Trans*-well inserts either uncoated (for migration assay) or coated (for invasion assay) with growth factor-reduced matrigel for 24 h. After incubation, cells were stained with Diff-Quick stain set (Fisher Scientific, Pittsburg, PA), and photographed under a Olympus CKX41 microscope.

### Sphere Forming Assay

EOC cells (500/well) were plated on Corning 24-well ultra-low attachment plates (Sigma-Aldrich) grown in serum free DMEM-F12 (ATCC) supplemented with B27 (Thermo Fisher), 20 ng/ml epidermal growth factor (Sigma-Aldrich), 0.4% bovine serum albumin (Sigma-Aldrich) and 4 μg/ml insulin (Sigma-Aldrich). Fresh medium was supplemented every 2 days. The spheroids were counted and photographed at day 14. For secondary spheroid formation, the primary spheroids were dissociated into single cells, and cultured on 24-well ultra-low attachement plates using spheroid culture medium for another 10 days.

### Statistical Analysis

Contingency table analysis and Chi square tests were used to study the relationship between clinico-pathological variables and protein expression. Progression-free survival curves were generated using the Kaplan–Meier method, with significance evaluated using the Mantel–Cox log-rank test. Multivariate analysis was performed using Cox proportional hazards regression model, after adjusting for clinic-pathological variables like age, histology, grade and stage of tumor. The limit of significance for all analyses was defined as *p* value of <0.05; two-sided tests were used in these calculations. The JMP11.0 (SAS Institute, Inc., Cary, NC) software package was used for data analyses.

For all functional studies, data presented are means ± SD of triplicates in an independent experiment, which was repeated for at least two times with the same results. Student t test (two-tailed) was performed for statistical significance with a *p* <0.05 used as the cut-off.

## Results

### KLF5 Expression and Clinico-pathological Associations

**FIGURE 1:**
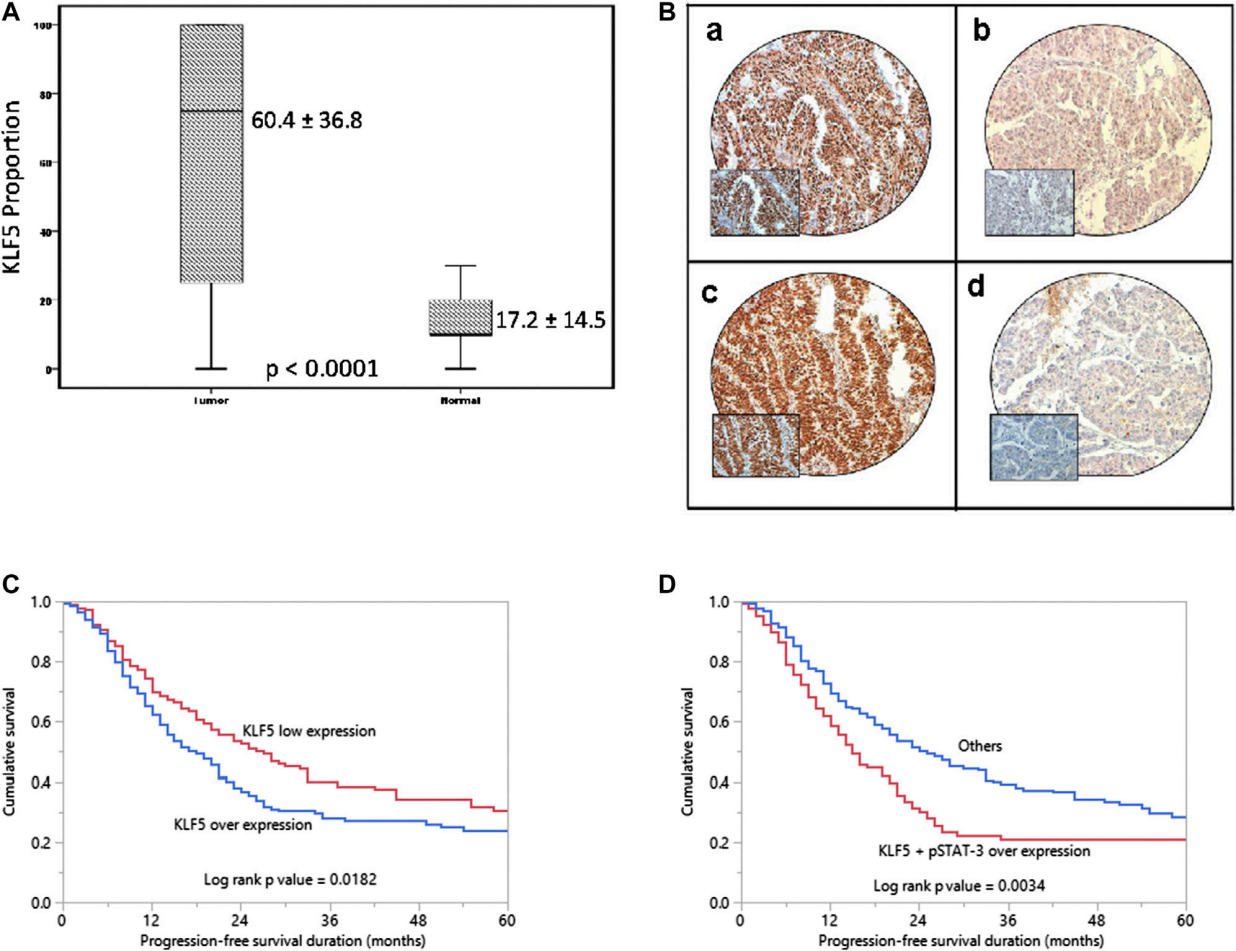
KLF5 protein expression and Progression-free survival curves in EOC. **(A)** A significant difference in expression levels was noted between normal ovary (*n* = 45) and EOC tissues (*n* = 425) (*p* < 0.0001). **(B)** Immunohistochemical analysis of KLF5 and pSTAT-3 expression in EOC TMA. An EOC array spot showing overexpression of KLF5 (a) and pSTAT-3 **(C)**. In contrast, another EOC tissue array spots showing low expression of KLF5 (b) and pSTAT-3 **(D)**. 20X/0.70 objective on an Olympus BX 51 microscope. (Olympus America Inc, Center Valley, PA, USA) with the inset showing a 40X 0.85 aperture magnified view of the same TMA spot. **(C)** Kaplan-Meier survival analysis for the prognostic significance of KLF5 expression in EOC showed that patients with overexpression of KLF5 had reduced progression-free survival at 5 years compared to tumors showing low expression of KLF5 (*p* = 0.0182). **(D)** EOC patients with co-expression of KLF5 and p-STAT3 had reduced progression-free survival at 5 years compared to tumors without co-expression of KLF5 and p-STAT3 (*p* = 0.0034).

KLF5 expression was determined in 425 EOC and 45 normal ovarian tissues by immunohistochemistry using TMA. We found KLF5 significantly upregulated in EOC tissues, compared to normal ovarian tissues (*p* <0.0001; Figure 1A). KLF5 overexpression was noted in 53.9% (229/425) of EOC cases and was associated with older age (*p* = 0.0070), larger tumor size (*p* = 0.0001), distant metastasis (*p* = 0.0001) and stage IV tumors (*p* < 0.0001). A significant association was also noted between KLF5 and pSTAT-3 (*p* < 0.0001) overexpression (Figure 1B). Interestingly, overexpression of KLF5 was significantly associated with progression-free survival in univariate analysis (*p* = 0.0182) (Table 2; Figure 1C). However, this significance was not seen on multivariate analysis after adjusting for confounding factors such as age, histology, grade and stage of tumor. We further analyzed the effect of KLF5 and p-STAT3 co-expression on clinical outcome and found that it was also significantly associated with poor progression-free survival in our cohort of EOC cases (*p* = 0.0034, Figure 1D).

**TABLE 2:** Clinicopathological associations of KLF5 protein expression in EOC.

	Total		KLF5 overexpression		KLF5 low expression		*p* value
	No	%	No	%	No	%	
No. of patients	425		229	53.9	196	46.1	
Age (years)							
≤50	215	50.6	102	47.4	113	52.6	0.0070
>50	210	49.4	127	60.5	83	39.5	
Histology type							
Serous	307	72.2	172	56.0	135	44.0	0.1145
Mucinous	62	14.7	26	41.9	36	58.1	
Endometrioid	38	8.9	18	47.4	20	52.6	
Clear cell	9	2.1	6	66.7	3	33.3	
Undifferentiated	9	2.1	7	77.8	2	22.2	
Histological grade							
Grade 1	89	21.6	44	49.4	45	50.6	0.3119
Grade 2	147	35.7	77	52.4	70	47.6	
Grade 3	176	42.7	103	58.5	73	41.5	
pT							
T1	89	21.2	31	34.8	58	65.2	0.0001
T2	38	9.0	19	50.0	19	50.0	
T3	293	69.8	176	60.1	117	39.9	
pN							
pN0	386	91.9	205	53.1	181	46.9	0.3318
pN1	34	8.1	21	61.8	13	38.2	
pM							
pM0	345	82.1	170	49.3	175	50.7	0.0001
pM1	75	17.9	56	74.7	19	25.3	
Stage							
I	83	20.0	23	27.7	60	72.3	<0.0001
II	24	5.8	11	45.8	13	54.2	
III	241	58.2	135	56.0	106	44.0	
IV	66	16.0	51	77.3	15	22.7	
pSTAT-3 IHC							
High	242	58.6	149	61.6	93	38.4	<0.0001
Low	171	41.4	71	41.5	100	58.5	
Progression free survival							
5 years							0.0182

### KLF5 Drives STAT3-Activation in EOC

**FIGURE 2:**
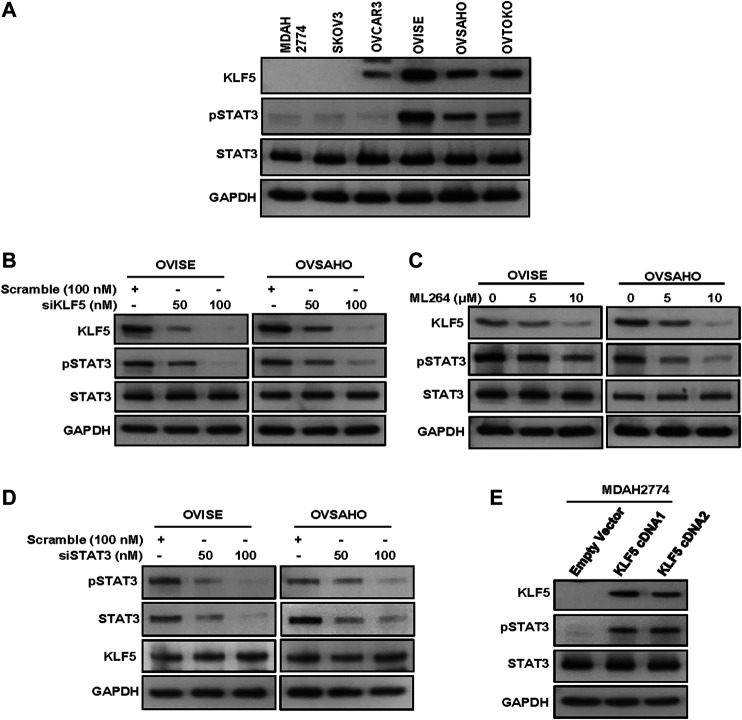
KLF5 drives STAT3-activation in EOC. **(A)** Basal expression of KLF5 and pSTAT3 in EOC cell lines. Proteins were isolated from six EOC cell lines and immunoblotted with antibodies against KLF5, pSTAT3, STAT3 and GAPDH. **(B)** Silencing of KLF5 inhibits STAT3 activation. EOC cells were transfected with scrambled siRNA and KLF5 siRNA (50 and 100 nM). After 48 hours, cells were lysed and proteins were immunoblotted with antibodies against KLF5, pSTAT3, STAT3 and GAPDH. **(C)** ML264 treatment down-regulates KLF5 expression and STAT3 activation in EOC cells. EOC cells were treated with indicated doses of ML264 for 48 h. After cell lysis, equal amounts of proteins were separated by SDS-PAGE, transferred to immobilon membrane, and immuno-blotted with antibodies against KLF5, pSTAT3, STAT3 and GAPDH as indicated. **(D)** Knockdown of STAT3 has no effect on KLF5 expression. EOC cells were transfected with scrambled siRNA and STAT3 siRNA (50 and 100 nM). After 48 h, cells were lysed and proteins were immunoblotted with antibodies against pSTAT3, STAT3, KLF5 and GAPDH. **(E)** Forced expression of KLF5 increases STAT3 activation. MDAH2774 cells were transfected with either empty vector or *KLF5* cDNA for 48 hours. Proteins were isolated and immunoblotted with antibodies against KLF5, pSTAT3, STAT3 and GAPDH for equal loading. All the experiments were repeated for at least two times with the same results.

Our clinical data showed that KLF5 was significantly associated with phospho-STAT3. To study this association *in vitro*, we first evaluated the basal expression of KLF5, phospho-STAT3 and total STAT3 in a panel of six cell lines by immuno-blotting. We identified concomitant expression of KLF5 and phospho-STAT3 in three EOC cell lines (OVISE, OVSAHO and OVTOKO), whereas cells with low or no expression of KLF5 (MDAH2774, SKOV3 and OVCAR3) showed low or no expression of activated STAT3 (Figure 2A and Supplementary Figure S1A). The basal expression of total STAT3 was similar in all of the tested cell lines. Next, we silenced KLF5 using specific siRNA in KLF5 expressing cells (OVISE and OVSAHO) and assessed the protein expression of KLF5, phospho-STAT3 and total STAT3 in these cells. As shown in Figure 2B, knockdown of KLF5 markedly down-regulated the expression of KLF5 and phospho-STAT3, whereas expression of total STAT3 remained unchanged in these cell lines (Supplementary Figure S1B). To confirm these above findings, we inhibited KLF5 using a specific KLF5 inhibitor, ML264 in KLF5 expressing cells and analyzed the expression of phospho-STAT3 and total STAT3 in these cells. Figure 2C shows that inhibition of KLF5 markedly down-regulated the expression of KLF5 and phospho-STAT3, whereas expression of total STAT3 remained unchanged after ML264 treatment in these cell lines (Supplementary Figure S1C). Next, we knockdown STAT3 to see the effect on KLF5 expression. As shown in Figure 2D, knockdown of STAT3 markedly down-regulated the expression of phospho-STAT3 and total STAT3, while KLF5 expression remained unchanged (Supplementary Figure S1D). To further verify the association between KLF5 and phospho-STAT3 *in vitro*, we overexpressed KLF5 in low expressing cell line (MDAH2774) and analyzed the phospho-STAT3 and total STAT3 expression. Figure 2E shows that forced expression of KLF5 dramatically increased the phospho-STAT3 and total STAT3 expression in MDAH2774 cell line (Supplementary Figure S1E). All these results indicate the strong association between KLF5 and phospho-STAT3 and that KLF5 functions upstream of phospho-STAT3 *in vitro*.

### Inhibition of KLF5 Decreases Invasion, Migration and Progression of Epithelial-To-Mesenchymal Transition in EOC Cells

**FIGURE 3:**
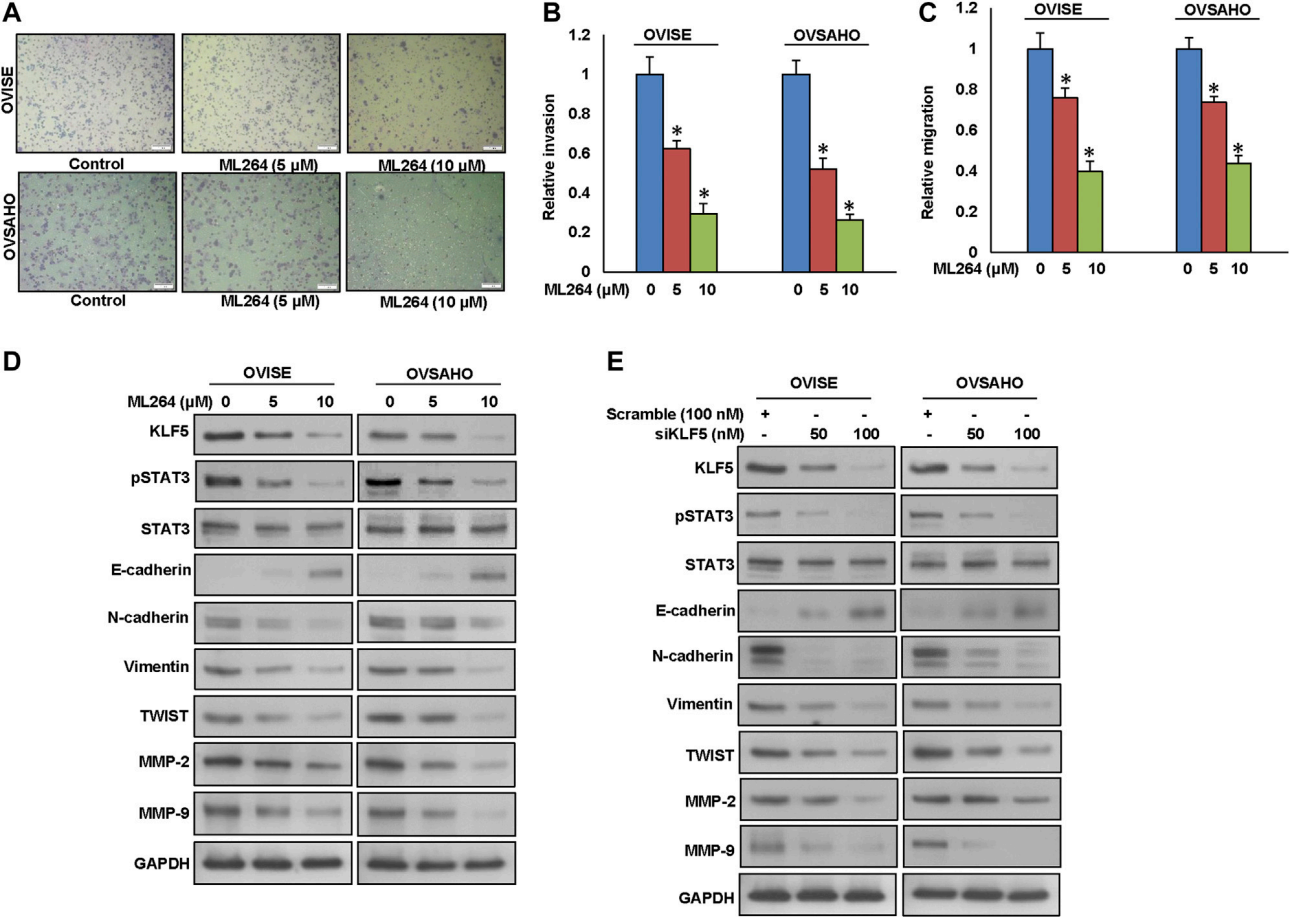
Inhibition of KLF5 decreases invasion, migration and progression of epithelial-to-mesenchymal transition in EOC cells. **(A**, **B)** KLF5 inhibition decreases the invasive capacity of EOC cells. EOC cells were pre-treated with universal caspase inhibitor, z-VAD/fmk (80 µM) for 3 h and subsequently treated with indicated doses of ML264 and seeded into the upper compartment of invasion chambers. The bottom chambers were filled with RPMI media. After 24 h incubation, invaded cells were fixed, stained and quantified. **(C)** KLF5 inhibition causes reduction in the migration capacity of EOC cells. EOC cells were pre-treated with universal caspase inhibitor, z-VAD/fmk (80 µM) for 3 h and subsequently treated with indicated doses of ML264 and seeded into the upper compartment of migration chambers. The bottom chambers were filled with RPMI media. After 24 h incubation, migrated cells were fixed, stained and quantified. **(D)** ML264 treatment down-regulates the expression of EMT markers in EOC cells. EOC cells were treated with indicated doses of ML264 for 48 h. After cell lysis, equal amounts of proteins were separated by SDS-PAGE, transferred to immobilon membrane, and immuno-blotted with antibodies against KLF5, pSTAT3, STAT3, E-cadherin, N-cadherin, Vimentin, Twist, MMP-2, MMP-9 and GAPDH as indicated. **(E)** Silencing of KLF5 down-regulates the expression of EMT markers in EOC cells. EOC cells were transfected with scrambled siRNA and KLF5 siRNA (50 and 100 nM). After 48 h, cells were lysed and proteins were immunoblotted with antibodies against KLF5, pSTAT3, STAT3, E-cadherin, N-cadherin, Vimentin, Twist, MMP-2, MMP-9 and GAPDH. Data presented in the bar graphs are the mean ± SD of triplicates in an independent experiments which was repeated for at least two times with the same results. *Indicates a statistically significant difference compared to control with *p* < 0.05. Western blot experiments were repeated at least two times with the same results.

Growing evidence indicates that overexpression of KLF5 is associated with EMT and metastasis (Zhang et al., 2013; Jia et al., 2016; Ma et al., 2018). Therefore, we investigated whether down-regulation of KLF5 would inhibit invasion, migration and progression of EMT in EOC cells. As expected, inhibition of KLF5 using ML264 significantly decreased invasion (Figures 3A,B) and migration (Figure 3C) of EOC cells. Furthermore, treatment with ML264 or KLF5 siRNA knockdown markedly down-regulated the expressions of KLF5, pSTAT3, N-cadherin, vimentin, Twist, MMP-2, and MMP-9 with an accompanying upregulation of E-cadherin expression in both EOC cells (Figures 3D,E and Supplementary Figures S2A,B). Conversely, forced expression of KLF5 in MDAH2774 cells (KLF5 low expressing cells) increased invasion (Supplementary Figure S3A) and migration (Supplementary Figure S3B) as well as upregulated the expression KLF5, pSTAT3, N-cadherin, vimentin, Twist, MMP-2, and MMP-9 with an accompanying downregulation of E-cadherin expression in these cells (Supplementary Figure S3C). However, total STAT3 expression remain unchanged in these cells.

To test the effect of STAT3 inhibition on invasion, migration and EMT progression, we silenced STAT3 using specific siRNA and analyzed invasion, migration and protein expressions of pSTAT3, STAT3, KLF5, E-cadherin, N-cadherin, Vimentin, TWIST, MMP-2 and MMP-9 in EOC cells. As shown in Supplementary Figure S4, silencing of STAT3 significantly decreased invasion (Supplementary Figure S4A) and migration (Supplementary Figure S4B) as well dramatically down-regulated pSTAT3, STAT3, N-cadherin, Vimentin, TWIST, MMP-2 and MMP-9 expressions and upregulated E-cadherin expression, while KLF5 level remains unchanged (Supplementary Figure S4C). These data show that KLF5-induced EMT progression is mediated via pSTAT3.

### ML264 Treatment Caused Inhibition of Cell Viability and Induction of Caspase-Dependent Apoptosis

**FIGURE 4:**
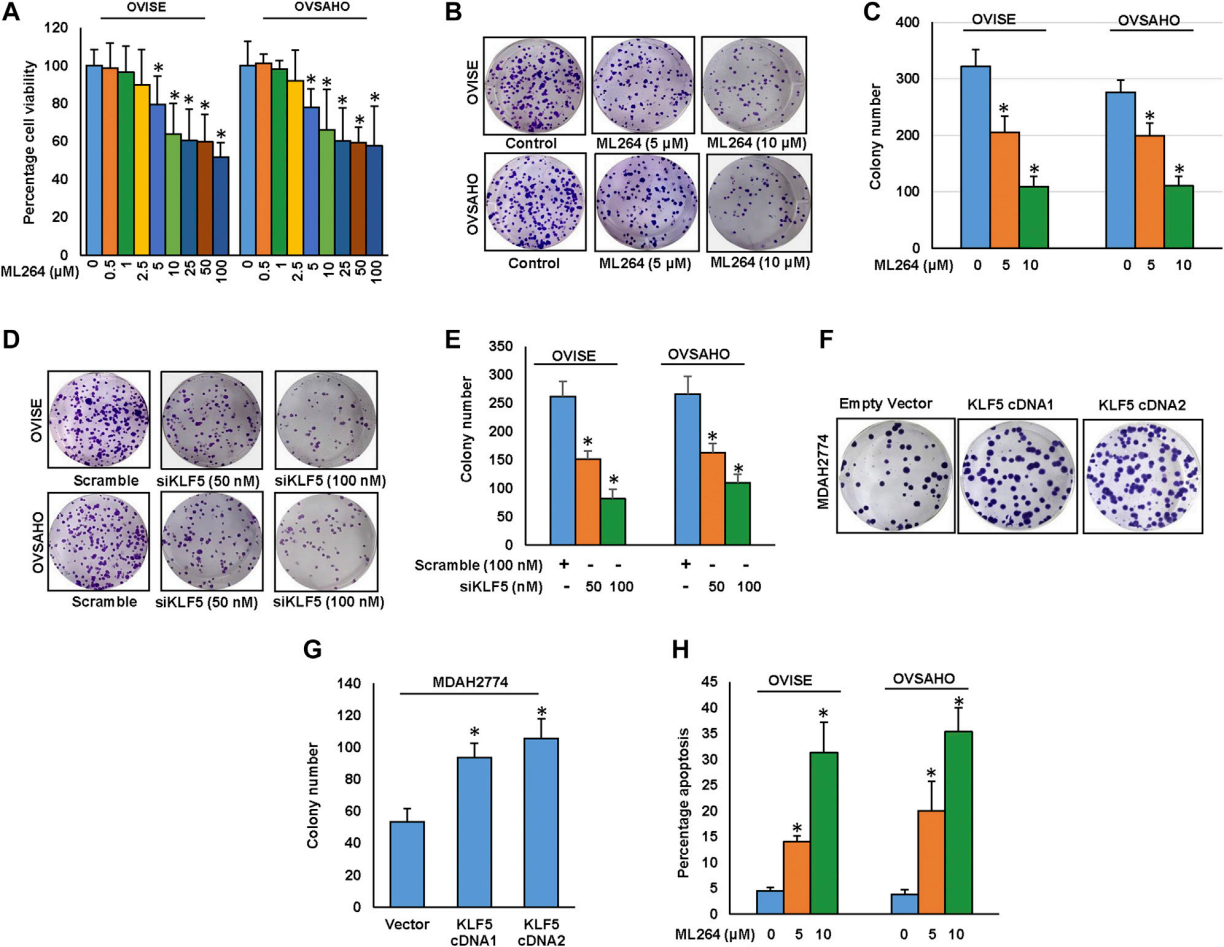
Downregulation of KLF5 inhibits EOC cell growth *in vitro.* **(A)** ML264 inhibits cell viability. EOC cells (10^4^) were incubated with indicated doses of ML264 for 48 hours. Cell viability was performed using MTT. **(B, C)** ML264 inhibited clonogenicity. EOC cells (8 × 10^2^) after ML264 treatment were seeded into each of two dishes (60 mm diameter), and grown for an additional 10 days, then stained with crystal violet and colonies were counted. **(D, E)** Knockdown of KLF5 decreases clonogenicity. EOC cells were transfected with scrambled siRNA and KLF5 siRNA (50 and 100 nM). After 48 h, cells (8 × 10^2^) were seeded into each of two dishes (60 mm diameter), and grown for an additional 10 days, then stained with crystal violet and colonies were counted. **(F-G)** Forced expression of KLF5 increases clonogenicity. MDAH2774 cells were transfected with either empty vector or *KLF5* cDNA. After 48 h, cells (8 × 10^2^) were seeded into each of two dishes (60 mm diameter), and grown for an additional 10 days, then stained with crystal violet and colonies were counted. **(H)** ML264 induces apoptosis in EOC cell lines. EOC cells were treated with indicated doses of ML264 for 48 h and cells were stained with fluorescein-conjugated annexin-V and propidium iodide (PI) and analyzed by flow cytometry. Data presented in the bar graphs are the mean ± SD of triplicates in an independent experiments which was repeated for at least two times with the same results. *Indicates a statistically significant difference compared to control with *p* < 0.05.

After analyzing the clinical data, we sought to determine whether targeting KLF5 expression in EOC cells be used as a viable therapeutic strategy to inhibit cell viability and induce apoptosis in addition to inhibition of invasion and migration. We therefore treated EOC cell lines; OVISE and OVSAHO with increasing doses of ML264 for 48 h and investigated cell viability by MTT assay. As shown in Figure 4A, there was a significant (*p* <0.05) dose-dependent inhibition of cell viability in both the cell lines. Next, we have treated EOC cells with indicated doses of ML264 and analyzed the colony number. Treatment of ML264 significantly (*p* <0.05) inhibited the clonogenicity of EOC cells as compared to untreated control (Figures 4B,C). In addition, knockdown of KLF5 significantly decreased the clonogenecity of EOC cells (Figures 4D,E). Conversely, forced expression of KLF5 in low expressing EOC cell (MDAH2774) increased the clonogenecity (Figures 4F,G). To determine whether inhibition of cell viability in KLF5 expressing EOC cells after treatment with ML264 was due to apoptosis, we treated both EOC cells with ML264 for 48 h and stained with annexin V and Propidium iodide and analyzed by flow cytometry. As shown in Figure 4H, there was an increase in apoptotic cells following treatment with ML264 in both the cell lines confirming that these cell death was due to apoptosis. Next, we sought to identify the apoptotic pathway activated by ML264 in inducing apoptosis.

**FIGURE 5:**
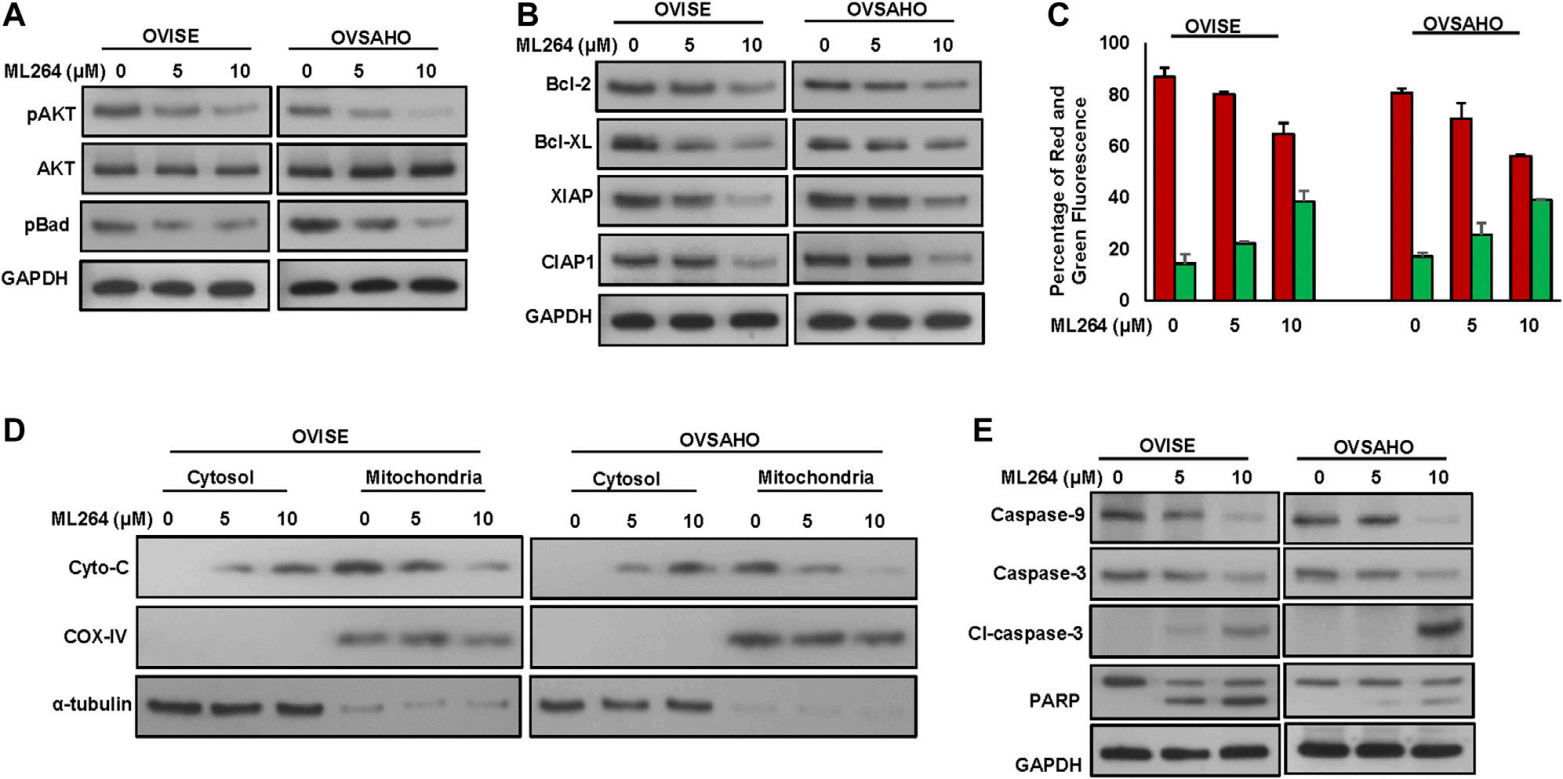
ML264 treatment causes inactivation of AKT and induces caspase-dependent apoptosis signaling cascade. **(A)** ML264 treatment causes inactivation of AKT and Bad proteins in EOC cells. EOC cells were treated with 5 and 10 μM ML264 for 48 h. Following treatment, cells were lysed and immunoblotted with antibodies against p-AKT, AKT, p-Bad and GAPDH. (**B)** ML264 treatment downregulates the expression of anti-apoptotic proteins and inhibitors of apoptosis in EOC cells. EOC cells were treated with indicated doses of ML264 for 48 h. After cell lysis, equal amounts of proteins were separated by SDS-PAGE, transferred to immobilon membrane, and immuno-blotted with antibodies against Bcl-2, Bcl-xl, XIAP, CIAP1 and GAPDH as indicated. (**C**) Estimation of mitochondrial membrane potential by flow cytometry. EOC cells were treated with 5 and 10 μM ML264 for 48 h. Following treatment, cells were stained with 10 μM JC1 and analysed by flow cytometry. (*n* = 3) (**D**) ML264-induced release of cytochrome c. EOC cells were treated with and without 5 and 10 μM ML264 for 48 h. Mitochondrial free, cytosolic fractions and mitochondrial extracts were isolated. Cell extracts were separated on SDS-PAGE, transferred to PVDF membrane, and immunoblotted with an antibody against cytochrome c, Cox IV and GAPDH. (**E**) Activation of caspases and cleavage of PARP induced by ML264 treatment in EOC cells. EOC cells were treated with and without 5 and 10 μM ML264 for 48 h. Cells were lysed, equal amount of proteins were separated on SDS-PAGE and immuno-blotted with antibodies against caspase-9, caspase-3, cleaved caspase-3, PARP and GAPDH, All the experiments were repeated twice with similar results. All the western blot experiments were repeated at least two times with the same results.

It has been previously shown that phosphorylation of AKT inhibits apoptosis via activation of Bad. We therefore examined the activation status of AKT and Bad in EOC cells following treatment with ML264 for 48 h. As shown in Figure 5A, there was dephosphorylation of AKT and Bad in ML264 treated EOC cells as determined by Western blotting (Supplementary Figure S5A). Dephosphorylated Bad forms a heterodimer with Bcl-2 and Bcl-xL, inactivating them, which in turn activate Bax or Bak, resulting in the release of cytochrome c and activation of caspase 9 and 3 (Siraj et al., 2018). Our data showed that there was down-regulation of expression of Bcl-2 and Bcl-xL following treatment with ML264 (Figure 5B and Supplementary Figure S5B). We then determined the effect of ML264 on Bax activation. We treated ML264 for different time points in EOC cell lines. We found evidence that Bax protein underwent conformational changes in both EOC cell lines tested (Supplementary Figure S5C). We next determined whether conformational changes in Bax protein caused change in mitochondrial membrane potential, the early event of activation of mitochondrial apoptotic pathway in EOC cells. We found that there was increased mitochondrial damage after ML264 treatment in both cell lines studied as depicted by an increase in green stained cells (apoptotic cells) as compared to red stained cells (normal cancer cells) (Figure 5C). Once the mitochondria are damaged, there is release of cytochrome c into cytosol thereby leading to activation and cleavage of caspase-9. To determine these findings, we treated EOC cells ML264 for 48 h and isolated cytosolic as well as mitochondrial extracts. As shown in Figure 5D, there was decrease in expression of cytochrome c in mitochondrial extracts following treatment with ML264 with concurrent increase of cytochrome c in the cytosolic compartment confirming mitochondrial damage and cytochrome c release. The release of cytochrome c into cytosol down-regulates Inhibitor of Apoptosis Proteins (IAPs), followed by the activation and cleavage of down-stream caspases and PARP. We examined the expression of IAPs; XIAP, and cIAP1 in EOC cells following treatment with ML264 for 48 h. There was down-regulation of the IAPs in both cell lines examined (Figure 5B). The treatment of ML264 also induced the cleavage of caspases-9, -3 and PARP in both the cell lines confirming caspase-dependent apoptosis in these cells (Figure 5E and Supplementary Figure S5B).

### Inhibition of KLF5 Decreased the Self-Renewal Ability of Spheroids Generated From EOC Cells

**FIGURE 6:**
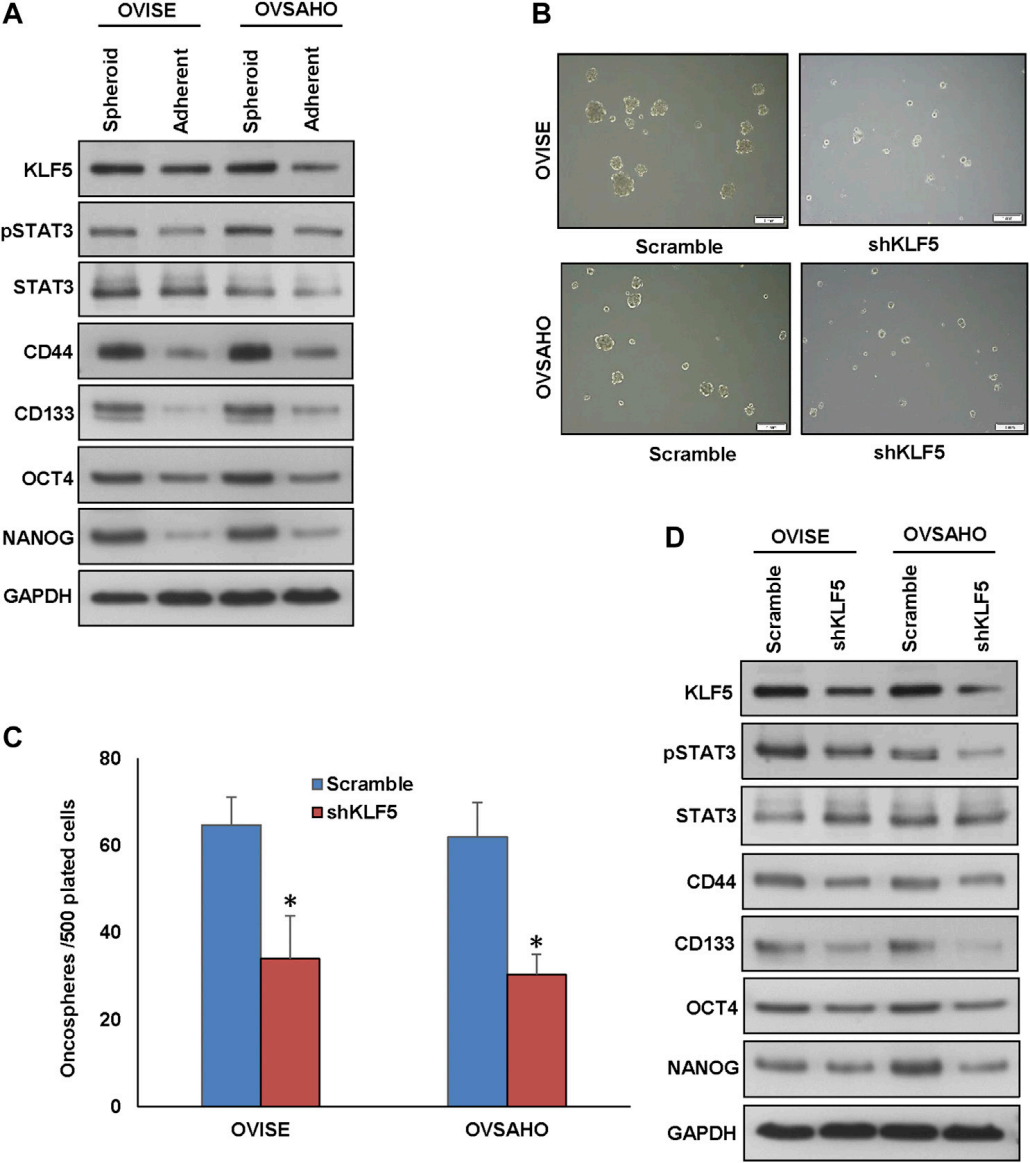
Inhibition of KLF5 decreases spheroid growth in EOC cells. **(A, B)** Isolation of spheroid-forming cells from EOC cells. Sphere forming assay was performed by culturing EOC cells (5 × 10^2^ cells/well) in sphere medium for 14 days in 24-well ultra-low attachment plates. Proteins were isolated from spheroid-forming cells and respective parental adherent cells and immunoblotted with antibodies against KLF5, pSTAT3, STAT3, CD44, CD133, NANOG, OCT4 and GAPDH. **(B, C)** Silencing of KLF5 inhibits self-renewal ability of spheroids. EOC cells were transfected with KLF5 shRNA and cells were subjected to sphere forming assay. Spheroids in the entire well were counted. **(D)** Silencing of KLF5 inhibits stemness of spheroids as confirmed by immunoblotting using stem cell markers. EOC cells were transfected with scramble or KLF5 shRNA’s and grown in sphere medium. Proteins were isolated from spheroids and immunoblotted with antibodies against KLF5, pSTAT3, STAT3, CD44, CD133, NANOG, OCT4 and GAPDH. Data presented in the bar graphs are the mean ± SD of triplicates in an independent experiments which was repeated for at least two times with the same results. *Indicates a statistically significant difference compared to control with *p* < 0.05. Western blot experiments were repeated at least two times with the same results.

KLF5 overexpression has been associated with stemness and self-renewal of cancer stem cells (Maehara et al., 2015). To test the role of KLF5 in spheroid growth in EOC, we generated spheroids from EOC cells and stemness of the spheroids were confirmed using stem cell markers (Figure 6A). There was a dramatic increase in the expression of stem cell markers, CD44, CD133, NANOG, and OCT4 in spheroids compared to respective adherent cells (Figure 6A and Supplementary Figure S6A). To ascertain the role of KLF5 in stemness maintenance in EOC, we silenced KLF5 in EOC cells and grown in spheroid medium. Interestingly, silencing of KLF5 significantly decreased the spheroid growth (Figures 6B,C) and stemness properties (Figure 6D and Supplementary Figure S6B). Above results, clearly indicate the role of KLF5 in stemness maintenance in EOC. Furthermore, forced expression of KLF5 in MDAH2774 cells (KLF5 low expressing cells) showed increased spheroid growth (Supplementary Figure S7A) and upregulated expression of KLF5, pSTAT3, CD44, CD133, NANOG, and OCT4 compared to empty vector transfected cells (Supplementary Figure S7B). However, total STAT3 expression remain unchanged in these cells.

To validate the role STAT3 activation on KLF5-induced spheroid growth, we silenced STAT3 using siRNA and analyzed the spheroid growth in EOC cells. As shown in Supplementary Figure S7C, knockdown of STAT3 significantly decreased the spheroid growth of both EOC cells tested. Above results, clearly indicate the role of KLF5 in stemness maintenance in EOC cells and is mediated via STAT3.

### ML264 Potentiates Anticancer Effect of Cisplatin in EOC Cells to Induce Efficient Apoptosis

**FIGURE 7:**
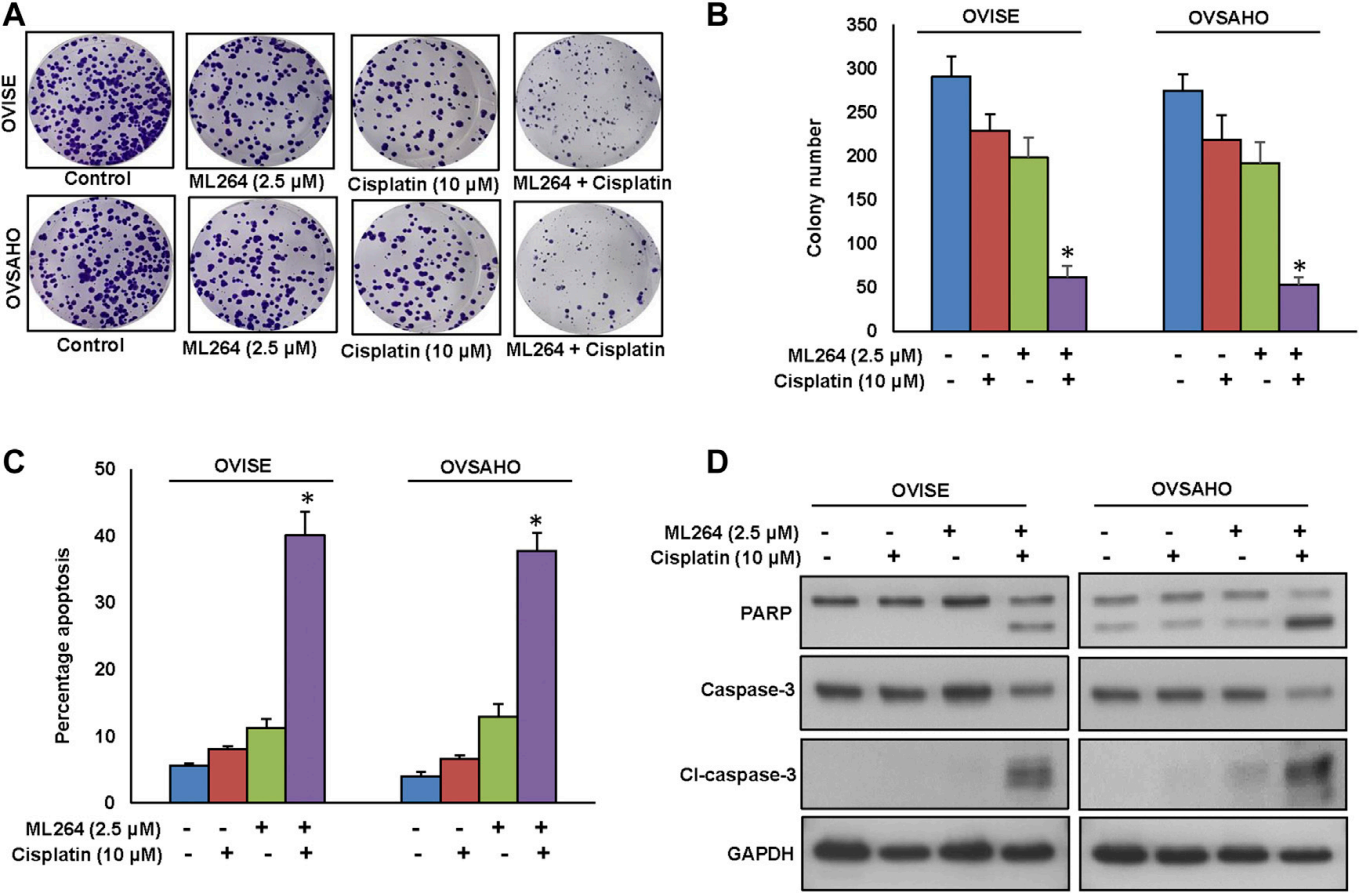
ML264 Potentiates anticancer effect of cisplatin in EOC cells to induce efficient apoptosis. **(A, B)** ML264 and cisplatin synergistically inhibits clonogenicity. EOC Cells (8 × 10^2^) after ML264 and cisplatin treatments were seeded into each of three dishes (60 mm diameter), and grown for an additional 10 days, then stained with crystal violet and colonies were counted. **(C)** ML264 potentiate cisplatin to induce apoptosis. EOC cells were treated with indicated doses of ML264 and cisplatin either alone or in combination for 48 h and cells were stained with flourescein-conjugated annexin-V and propidium iodide (PI) and analyzed by flow cytometry. **(D)** ML264 potentiate cisplatin to induce the cleavage of caspase-3 and PARP. EOC cells were treated with indicated doses of ML264 and cisplatin either alone or in combination for 48 h. Proteins were isolated and were immunoblotted with antibodies against caspase-3, cleaved caspase-3, PARP and GAPDH. Data presented in the bar graphs are the mean ± SD of triplicates in an independent experiments which was repeated for at least two times with the same results. *Indicates a statistically significant difference compared to control with *p* < 0.05. Western blot experiments were repeated at least two times with the same results.

Cisplatin is an important therapeutic tool in the combat against several solid neoplasms, including ovarian cancers, but its therapeutic effects are hampered by drug resistance (Wang et al., 2019). Combination therapy is an effective strategy to overcome the drug resistance (Nacarelli et al., 2020). We used multiple combinations of ML264 and cisplatin to determine the appropriate dose required to induce a synergistic apoptotic response in EOC cells. Using Calcusyn software and the Chou and Talalay methodology (Chou, 2010), we found that 2.5 µM ML264 and 10 μM cisplatin in combination had a combination index of 0.317 ad 0.603 in OVISE and OVSAHO cell lines respectively, indicating a synergistic response (Supplementary Figures S8 and SB). Using these doses, we treated OVISE and OVSAHO cell lines for 48 h and performed clonogenicity assay. There was a significant inhibition of colony number in both cell lines (Figures 7A,B). The co-treatment of sub-optimal doses of ML264 and cisplatin synergistically induced apoptosis in both EOC cell lines tested (Figure 7C). Finally, combination treatment of EOC cell lines with suboptimal doses of ML264 and cisplatin induced the cleavage of caspases-3 and PARP (Figure 7D and Supplementary Figure S10). These data clearly suggest that ML264 potentiate the effect of cisplatin to induce apoptosis in EOC cells.

## Discussion

The low 5 years overall survival of EOC despite multimodal treatment, highlight the importance of identifying more effective treatment approach to improve patient outcome. In this study, 425 Middle Eastern EOC were evaluated for KLF5 protein expression after tissue microarray was constructed. KLF5 overexpression was seen in more than 53% of the patient samples and was significantly associated with aggressive clinico-pathological criteria including larger tumor size, distant metastasis, stage IV tumors and poor patient survival. Several previous studies in many organ sites have reported the presence of KLF5 protein expression in patient samples (Tong et al., 2006; Ma et al., 2018; Li et al., 2019). Others have identified a strong association of KLF5 overexpression with aggressive markers and poor outcome (Soon et al., 2011; Yang et al., 2017). Our findings reveal that KLF5 might be a potential prognostic marker in Middle Eastern EOC.

A previous study showed that KLF5 disruption reduces STAT3 activation and tumor growth of pancreatic ductal adenocarcinoma *in vivo* (He et al., 2018). This prompts us to study the association of KLF5 with phospho-STAT3 in EOC patient samples. Our clinical data showed that KLF5 protein expression was significantly associated with phospho-STAT3 protein expression. Importantly, the co-expression of KLF5 and p-STAT3 is significantly associated with poor progression-free survival in our cohort of EOC cases. To test the association of KLF5 with phospho-STAT3, *in vitro*, we assessed the basal expression of KLF5 and phospho-STAT3 in a panel of six cell lines by immunoblot. Based on KLF5 and phospho-STAT3 expression, we identified three EOC cell lines that had concomitant expression of KLF5 and phospho-STAT3, as well as another three cell lines that had negligible expression of KLF5 and phospho-STAT3. Silencing of KLF5 decreased the expression of phospho-STAT3, while KLF5 was not affected by STAT3 inhibition. Furthermore, forced expression of KLF5 dramatically increased the phospho-STAT3 expression in KLF5 low expressing cell lines. Collectively, these data demonstrate an association between KLF5 and phospho-STAT3, and that KLF5 functions upstream of phospho-STAT3.

In order to determine the functional role of KLF5 in EOC cell lines, we manipulated KLF5 expression in EOC cell lines. As expected, forced expression of KLF5 enhanced the cell growth while KLF5 silencing decreased the growth of EOC cells. Due to KLF5 overexpression correlating with aggressive clinico-pathological parameters and poor outcome in EOC patients, we sought to investigate whether targeting KLF5 expression, using ML264, would be a viable therapeutic strategy to inhibit cell growth and induce apoptosis in EOC cells. Pharmacological inhibition of KLF5 dramatically decreased cell viability and induced mitochondrial dependent apoptosis in EOC cells. This finding is consistent with previous studies in osteosarcoma (Huang et al., 2020) and colorectal cancer (de Sabando et al., 2016). KLF5 has been associated with cancer cell invasion, migration and EMT progression (Zhang et al., 2013; Ma et al., 2017). Sun L et al., found that the function of KLF5 in EMT and migration of liver cancer cells depended on the p53 status (Sun et al., 2020). KLF5 promotes migration and invasion by upregulating the transcription of TNFAIP2, a tumor necrosis factor-α (TNFα)-induced gene in breast cancer cells (Jia et al., 2016). In osteosarcoma cell lines, ML264 treatment found to inhibits the activation of STAT3 and proteins associated with EMT by decreasing KLF5 expression (Huang et al., 2020). Inhibition of KLF5 using siRNA or ML264 markedly decreased invasion, migration and EMT progression in EOC cells. Thus, indicating that the inhibition of KLF5 induces apoptosis, and decreases the metastatic potential of EOC cells.

Chemotherapy resistance is a major drawback of ovarian cancer treatment (Pokhriyal et al., 2019), where ovarian cancer stem cells have been shown to survive after conventional chemotherapy (Motohara and Katabuchi, 2019). Subsequently, this selected population of cancer stem cells gives rise to chemo-resistant recurrent tumors at metastatic sites (Yap et al., 2009; Motohara and Katabuchi, 2019). In concordance with a previous study in hepatocellular carcinoma (Maehara et al., 2015), we found that KLF5 inhibition dramatically decreased the spheroid growth and downregulated the expression of stem cell markers such as CD44, CD133, NANOG and OCT4 in EOC cells. These findings demonstrate the pivotal role of KLF5 in regulation of cancer stem-like cells in EOC cells. We also demonstrated that treatment with suboptimal doses of ML264, potentiated cisplatin-induced apoptosis in EOC cells, which is in line with a previous report in prostate cancer (Jia et al., 2019).

In summary, our results demonstrate KLF5 overexpression in Middle Eastern EOC patient samples, which was further significantly associated with aggressive clinico-pathological parameters and poor outcome. These data clearly demonstrate that KLF5 plays a significant role in EOC pathogenesis. Functional analysis showed that inhibition of KLF5 decreased invasion, migration and EMT progression in EOC cells. Additionally, downregulation of KLF5 potentiated cisplatin-induced apoptosis in EOC cells. Exclusive or combinatory pharmacological inhibition of KLF5, with conventional chemotherapeutic agents, such as cisplatin may be a promising therapeutic strategy for EOC patients exhibiting aggressive subtypes.

## Data Availability Statement

The original contributions presented in the study are included in the article/Supplementary Material, further inquiries can be directed to the corresponding author.

## Ethics Statement

The studies involving human participants were reviewed and approved by Institutional Review Board of the King Faisal Specialist Hospital and Research Center. Written informed consent for participation was not required for this study in accordance with the national legislation and the institutional requirements.

## Author Contributions

AS and PP designed, performed experiments and wrote the manuscript. SD performed experiments and data analysis. SP prepared the TMA and conducted all the immunohistochemistry experiments and scoring of IHC spots. ST and KA performed experiments, SS wrote the manuscript, IA and FA contributed samples and analyzed clinical data, KA designed, implemented the study, wrote and critically reviewed the manuscript. This is to confirm that all authors read and approved the final manuscript.

## Conflict of Interest

The authors declare that the research was conducted in the absence of any commercial or financial relationships that could be construed as a potential conflict of interest.

## Glossary

EOC: Epithelial ovarian cancer
KLF5: Kruppel Like Factor 5
STAT3: Signal transducer and activator of transcription 3
IHC: Immunohistochemistry
TMA: Tissue microarray
ATCC: American type culture collection
FBS: Fetal bovine serum
MTT: 3-(4,5-dimethylthiazol-2-yl)-2,5-diphenyltetrazolium bromide
EMT: Epithelial–mesenchymal transition
MMP: Matrix metalloproteinase
XIAP: X-linked inhibitor of apoptosis protein
Bcl-2: B-cell lymphoma 2
Bcl-xl: B-cell lymphoma-extra-large
cIAP1: Cellular inhibitor of apoptosis protein 1
PARP: Poly (ADP-ribose) polymerase

## References

[B1] Bu R. Siraj A. K. Divya S. P. Kong Y. Parvathareddy S. K. Al‐Rasheed M.(2018). Telomerase reverse transcriptase mutations are independent predictor of disease‐free survival in M iddle E astern papillary thyroid cancer. Int. J. Cancer 142, 2028–2039. 10.1002/ijc.31225 29266240

[B2] Chou T.-C. (2010). Drug combination studies and their synergy quantification using the Chou–Talalay method. Cancer Res. 70, 440–446. 10.1158/0008-5472.can-09-1947 20068163

[B3] De Sabando A. R. Wang C. He Y. García-Barros M. Kim J. Shroyer K. R. (2016). ML264, a novel small-molecule compound that potently inhibits growth of colorectal cancer. Mol. Cancer Therapeut. 15, 72–83. 10.1158/1535-7163.mct-15-0600 PMC4707060 26621868

[B4] Dong Z. Yang L. Lai D. (2013). KLF 5 strengthens drug resistance of ovarian cancer stem‐like cells by regulating survivin expression. Cell Prolif. 46, 425–435. 10.1111/cpr.12043 23869764 PMC6496892

[B5] Ghaleb A. M., Yang V. W. (2008). The pathobiology of Krüppel-like factors in colorectal cancer. Curr. Color. Cancer Rep. 4, 59–64. 10.1007/s11888-008-0011-4 PMC2394726 18504508

[B6] Guanizo A. C. Fernando C. D. Garama D. J. Gough D. J. (2018). STAT3: a multifaceted oncoprotein. Growth Factors 36, 1–14. 10.1080/08977194.2018.1473393 29873274

[B7] Hayashi S. Manabe I. Suzuki Y. Relaix F. Oishi Y. (2016). Klf5 regulates muscle differentiation by directly targeting muscle-specific genes in cooperation with MyoD in mice. Elife 5, e17462 10.7554/elife.17462.032 27743478 PMC5074804

[B8] He P. Yang J. W. Yang V. W. Bialkowska A. B. (2018). Krüppel-like factor 5, increased in pancreatic ductal adenocarcinoma, promotes proliferation, acinar-to-ductal metaplasia, pancreatic intraepithelial neoplasia, and tumor growth in mice. Gastroenterology 154, 1494–1508. 10.1053/j.gastro.2017.12.005 29248441 PMC5880723

[B9] Huang H. Han Y. Chen Z. Pan X. Yuan P. Zhao X. (2020). ML264 inhibits osteosarcoma growth and metastasis via inhibition of JAK2/STAT3 and WNT/β‐catenin signalling pathways. J. Cell Mol. Med. 12, 33–37. 10.1111/jcmm.15226 PMC7214147 32285603

[B10] Jia J. Zhang H.-B. Shi Q. Yang C. Ma J.-B. Jin B. (2019). KLF5 downregulation desensitizes castration-resistant prostate cancer cells to docetaxel by increasing BECN1 expression and inducing cell autophagy. Theranostics 9, 5464 10.7150/thno.33282 31534497 PMC6735397

[B11] Jia L. Zhou Z. Liang H. Wu J. Shi P. Li F. (2016). KLF5 promotes breast cancer proliferation, migration and invasion in part by upregulating the transcription of TNFAIP2. Oncogene 35, 2040–2051. 10.1038/onc.2015.263 26189798

[B12] Levy D. E., Inghirami G. (2006). STAT3: a multifaceted oncogene. Proc. Natl. Acad. Sci. Unit. States Am. 103, 10151–10152. 10.1073/pnas.0604042103 PMC1502425 16801534

[B13] Li Q. Dong Z. Zhou F. Cai X. Gao Y. Wang L.-W. (2014). Krüppel-like factor 5 promotes lung tumorigenesis through upregulation of Sox4. Cell. Physiol. Biochem. 33, 1–10. 10.1159/000356645 24401325

[B14] Li Y. Kong R. Chen H. Zhao Z. Li L. Li J. (2019). Overexpression of KLF5 is associated with poor survival and G1/S progression in pancreatic cancer. Aging 11, 5035 10.18632/aging.102096 31327760 PMC6682527

[B15] Li Y. Sui X. Hu X. Hu Z. (2018). Overexpression of KLF5 inhibits puromycin-induced apoptosis of podocytes. Mol. Med. Rep. 18, 3843–3849. 10.3892/mmr.2018.9366 30106142 PMC6131625

[B16] Liang R. Chen X. Chen L. Wan F. Chen K. Sun Y. (2020). STAT3 signaling in ovarian cancer: a potential therapeutic target. J. Cancer 11, 837 10.7150/jca.35011 31949487 PMC6959025

[B17] Ma D. Chang L.-Y. Zhao S. Zhao J.-J. Xiong Y.-J. Cao F.-Y., et al. (2017). KLF5 promotes cervical cancer proliferation, migration and invasion in a manner partly dependent on TNFRSF11a expression. Sci. Rep. 7, 1–13. 10.1038/s41598-017-15979-1 29146991 PMC5691198

[B18] Ma Y. Wang Q. Liu F. Ma X. Wu L. Guo F. (2018). KLF5 promotes the tumorigenesis and metastatic potential of thyroid cancer cells through the NF-κB signaling pathway. Oncol. Rep. 40, 2608–2618. 10.3892/or.2018.6687 30226614 PMC6151893

[B19] Maehara O. Sato F. Natsuizaka M. Asano A. Kubota Y. Itoh J. (2015). A pivotal role of Krüppel-like factor 5 in regulation of cancer stem-like cells in hepatocellular carcinoma. Cancer Biol. Ther. 16, 1453–1461. 10.1080/15384047.2015.1070992 26176896 PMC4846134

[B20] Mori A. Moser C. Lang S. A. Hackl C. Gottfried E. Kreutz M. (2009). Up-regulation of Krüppel-like factor 5 in pancreatic cancer is promoted by interleukin-1β signaling and hypoxia-inducible factor-1α. Mol. Canc. Res. 7, 1390–1398. 10.1158/1541-7786.mcr-08-0525 19671674

[B21] Motohara T., Katabuchi H. (2019). Ovarian cancer stemness: biological and clinical implications for metastasis and chemotherapy resistance. Cancers 11, 907 10.3390/cancers11070907 PMC6678827 31261739

[B22] Nacarelli T. Fukumoto T. Zundell J. A. Fatkhutdinov N. Jean S. Cadungog M. G. (2020). NAMPT inhibition suppresses cancer stem-like cells associated with therapy-induced senescence in ovarian cancer. Cancer Res 80, 890–900. 10.1158/0008-5472.can-19-2830 31857293 PMC7024650

[B23] Nakajima Y. Akaogi K. Suzuki T. Osakabe A. Yamaguchi C. Sunahara N. (2011). Estrogen regulates tumor growth through a nonclassical pathway that includes the transcription factors ERβ and KLF5. Sci. Signal. 4, ra22 10.1126/scisignal.2001551 21487105

[B24] Pokhriyal R. Hariprasad R. Kumar L. Hariprasad G. (2019). Chemotherapy resistance in advanced ovarian cancer patients. Biomark. Cancer 11, 1179299 10.1177/1179299x19860815 PMC6613062 31308780

[B25] Saini U. Naidu S. Elnaggar A. C. Bid H. K. Wallbillich J. J. Bixel K. (2017). Elevated STAT3 expression in ovarian cancer ascites promotes invasion and metastasis: a potential therapeutic target. Oncogene 36, 168–181. 10.1038/onc.2016.197 27292260 PMC5338638

[B26] Siddiquee K. a. Z. Turkson J. (2008). STAT3 as a target for inducing apoptosis in solid and hematological tumors. Cell Res. 18, 254–267. 10.1038/onc.2016.197 18227858 PMC2610254

[B27] Siraj A. Bavi P. Abubaker J. Jehan Z. Sultana M. Al‐Dayel F. (2007). Genome‐wide expression analysis of Middle Eastern papillary thyroid cancer reveals c‐MET as a novel target for cancer therapy. J. Pathol. 213, 190–199. 10.1038/cr.2008.1810.1002/path.2215 17703498

[B28] Siraj A. K. Pratheeshkumar P. Parvathareddy S. K. Qadri Z. Thangavel S. Ahmed S. (2018). FoxM1 is an independent poor prognostic marker and therapeutic target for advanced Middle Eastern breast cancer. Oncotarget 9, 17466 10.1002/path.221510.18632/oncotarget.24739 29707121 PMC5915129

[B29] Soon M.-S. Hsu L.-S. Chen C.-J. Chu P.-Y. Liou J.-H. Lin S.-H. (2011). Expression of Krűppel-like factor 5 in gastric cancer and its clinical correlation in Taiwan. Virchows Arch. 459, 161 10.1007/s00428-011-1111-0 21732124

[B30] Sun L. Zhou X. Li Y. Chen W. Wu S. Zhang B. (2020). KLF5 regulates epithelial-mesenchymal transition of liver cancer cells in the context of p53 loss through miR-192 targeting of ZEB2. Cell Adhes. Migrat. 14, 72–74. 10.1080/19336918.2020.1826216 PMC7553557 32965165

[B31] Takagi K. Miki Y. Onodera Y. Nakamura Y. Ishida T. Watanabe M. (2012). Krüppel-like factor 5 in human breast carcinoma: a potent prognostic factor induced by androgens. Endocr. Relat. Canc. 19, 741–750. 10.1530/erc-12-0017 22936544

[B32] Tong D. Czerwenka K. Heinze G. Ryffel M. Schuster E. Witt A. (2006). Expression of KLF5 is a prognostic factor for disease-free survival and overall survival in patients with breast cancer. Clin. Canc. Res. 12, 2442–2448. 10.1158/1078-0432.ccr-05-0964 16638850

[B33] Ushijima K. (2010). Treatment for recurrent ovarian cancer—at first relapse. J. Oncol. 10, 13–17. 10.1155/2010/497429 PMC2801501 20066162

[B34] Wang Z. Deng Z. Zhu G. (2019). Emerging platinum (IV) prodrugs to combat cisplatin resistance: from isolated cancer cells to tumor microenvironment. Dalton Trans. 48, 2536–2544. 10.1039/c8dt03923b 30633263

[B35] Wu C.-J. Sundararajan V. Sheu B.-C. Huang R. Y.-J. Wei L.-H. (2020). Activation of STAT3 and STAT5 signaling in epithelial ovarian cancer progression: mechanism and therapeutic opportunity. Cancers 12, 24 10.3390/cancers12010024 PMC7017004 31861720

[B36] Yang T. Chen M. Yang X. Zhang X. Zhang Z. Sun Y. (2017). Down-regulation of KLF5 in cancer-associated fibroblasts inhibit gastric cancer cells progression by CCL5/CCR5 axis. Cancer Biol. Ther. 18, 806–815. 10.1080/15384047.2017.1373219 28934010 PMC5678703

[B37] Yap T. A. Carden C. P. Kaye S. B. (2009). Beyond chemotherapy: targeted therapies in ovarian cancer. Nat. Rev. Cancer 9, 167–181. 10.1038/nrc2583 19238149

[B38] Yoshikawa T. Miyamoto M. Aoyama T. Soyama H. Goto T. Hirata J. (2018). JAK2/STAT3 pathway as a therapeutic target in ovarian cancers. Oncology Lett. 15, 5772–5780. 10.3892/ol.2018.8028 PMC5840758 29545902

[B39] Zhang B. Zhang Z. Xia S. Xing C. Ci X. Li X. (2013). KLF5 activates microRNA 200 transcription to maintain epithelial characteristics and prevent induced epithelial-mesenchymal transition in epithelial cells. Mol. Cell Biol. 33, 4919–4935. 10.1128/mcb.00787-13 24126055 PMC3889554

[B40] Zhang H.-F., Lai R. (2014). STAT3 in cancer—friend or foe? Cancers 6, 1408–1440. 10.3390/cancers6031408 24995504 PMC4190548

