# Supplementary material for: Krupple-Like Factor 5 is a Potential Therapeutic Target and Prognostic Marker in Epithelial Ovarian Cancer
Source: Front Pharmacol. 2020 Dec 3;11:598880. doi: 10.3389/fphar.2020.598880 (PMC7793801; doi:10.3389/fphar.2020.598880)
Supplement: Supplementary file 1 [file datasheet1.pdf]

Supplementary Figure 1

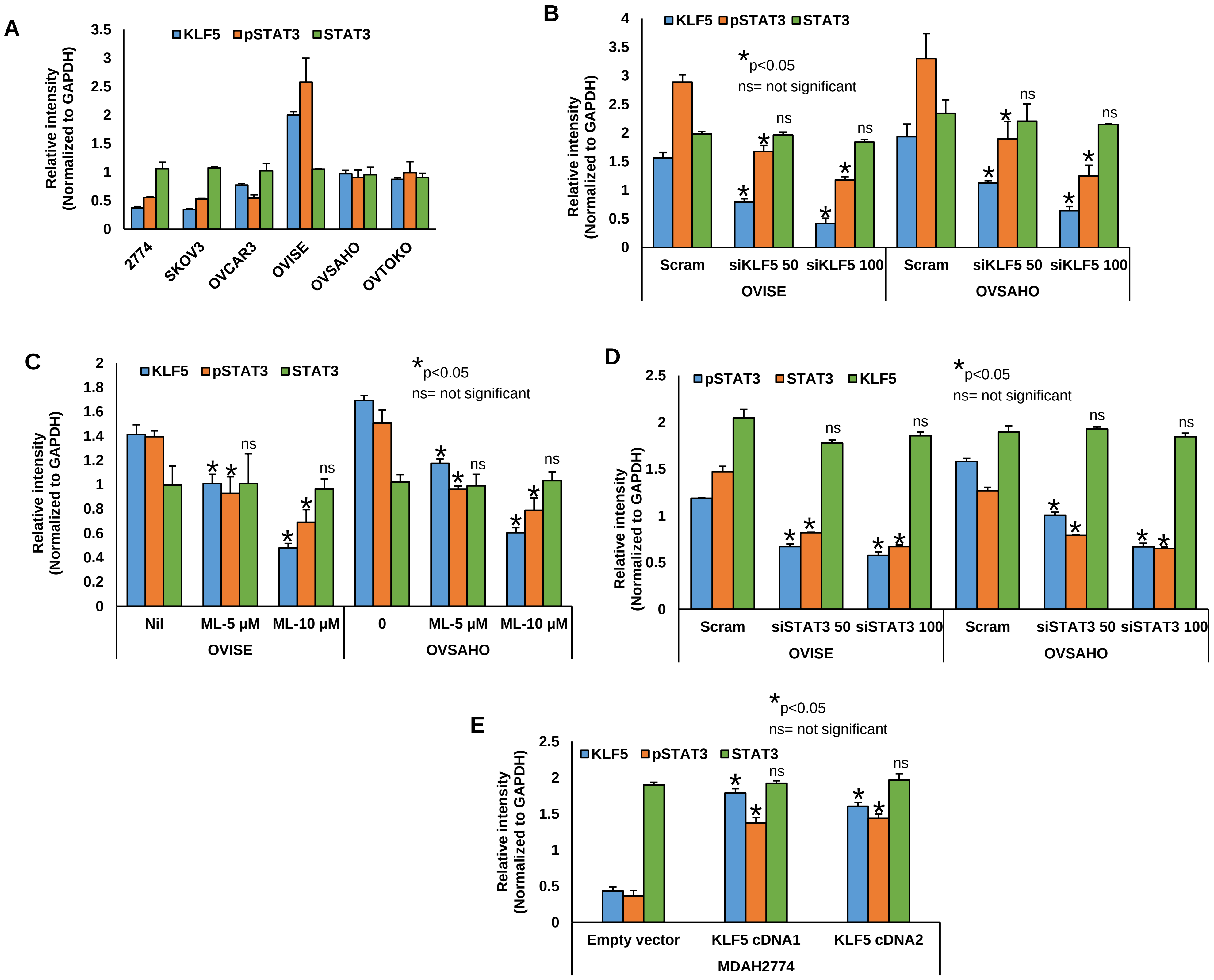

Supplementary Figure 2

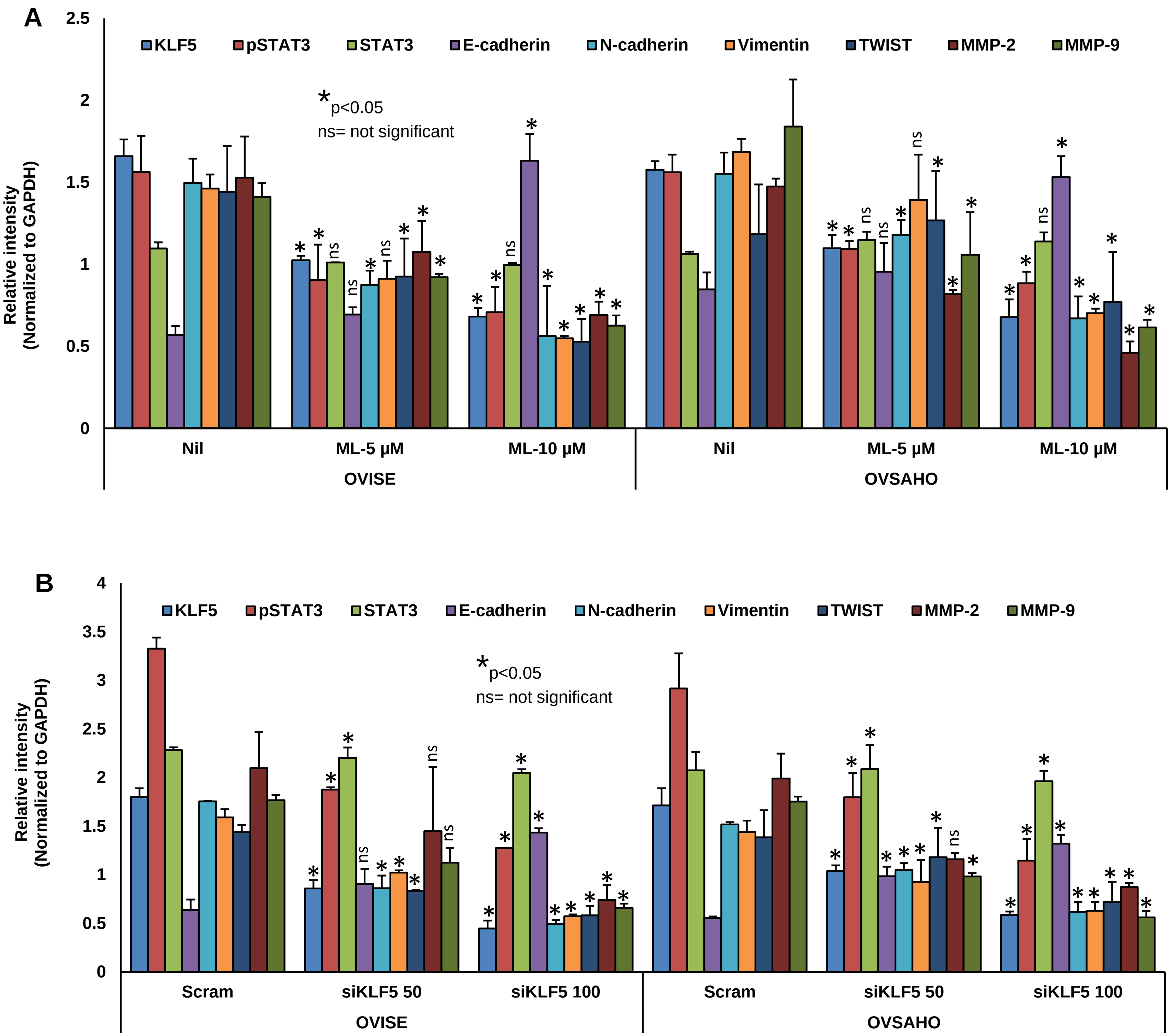

Supplementary Figure 3

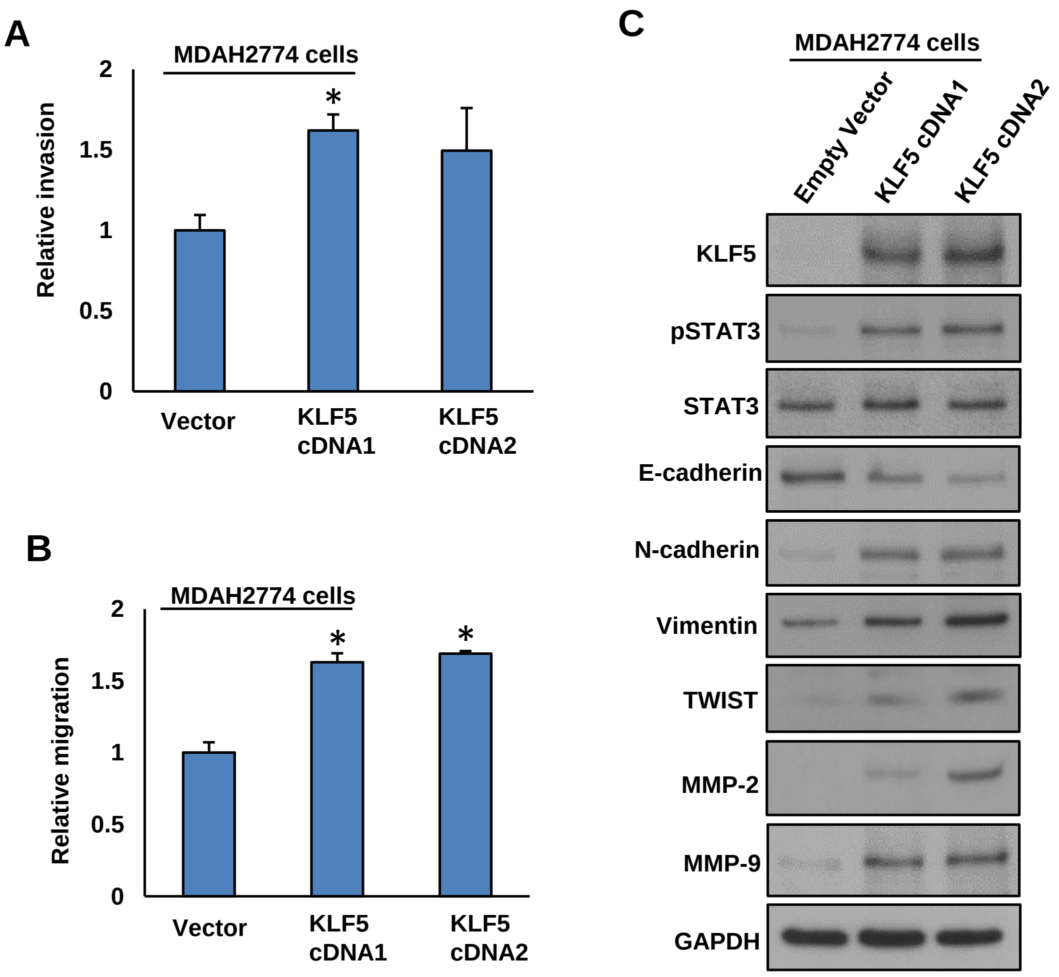

Supplementary Figure 4

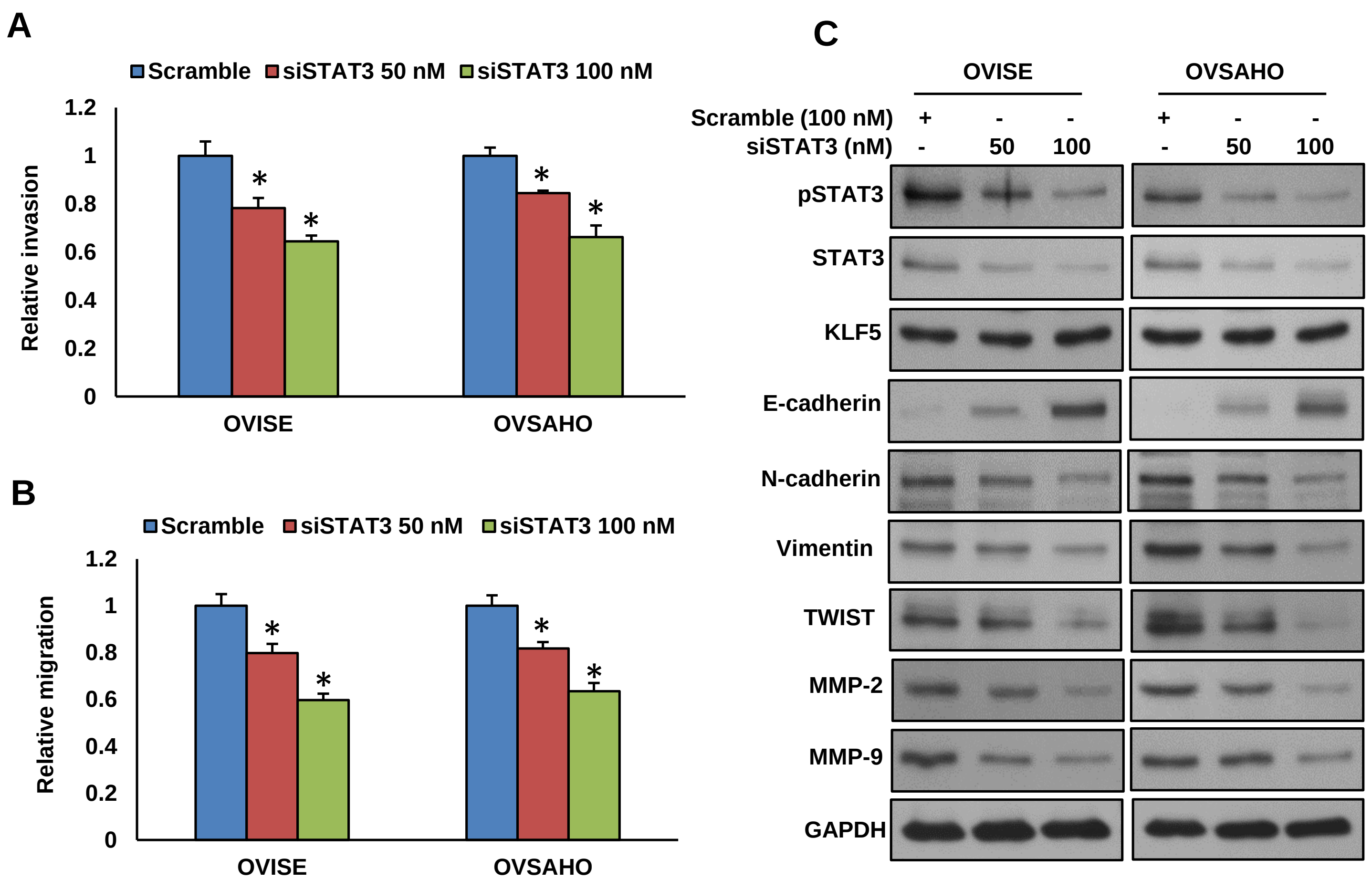

Supplementary Figure 5

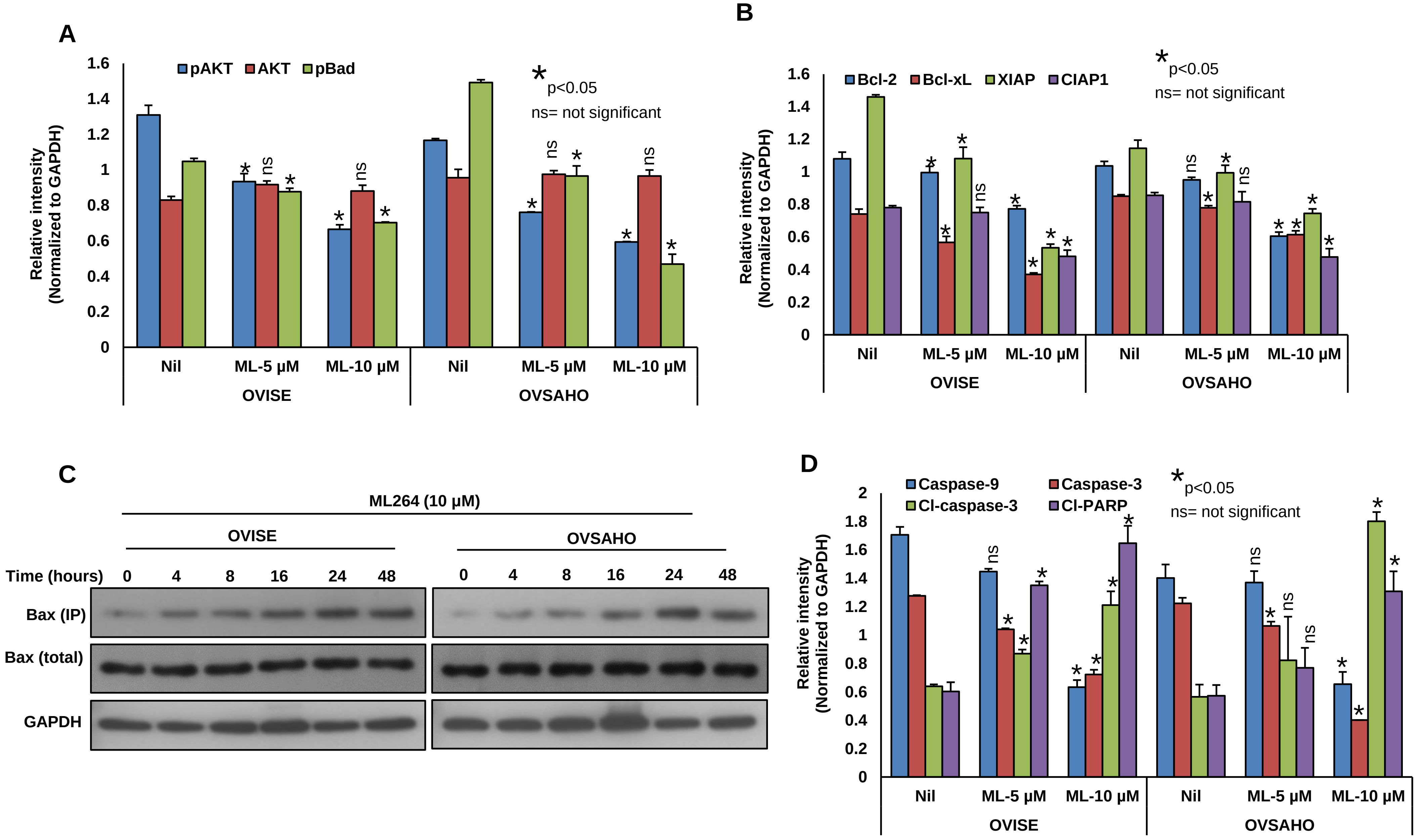

Supplementary Figure 6

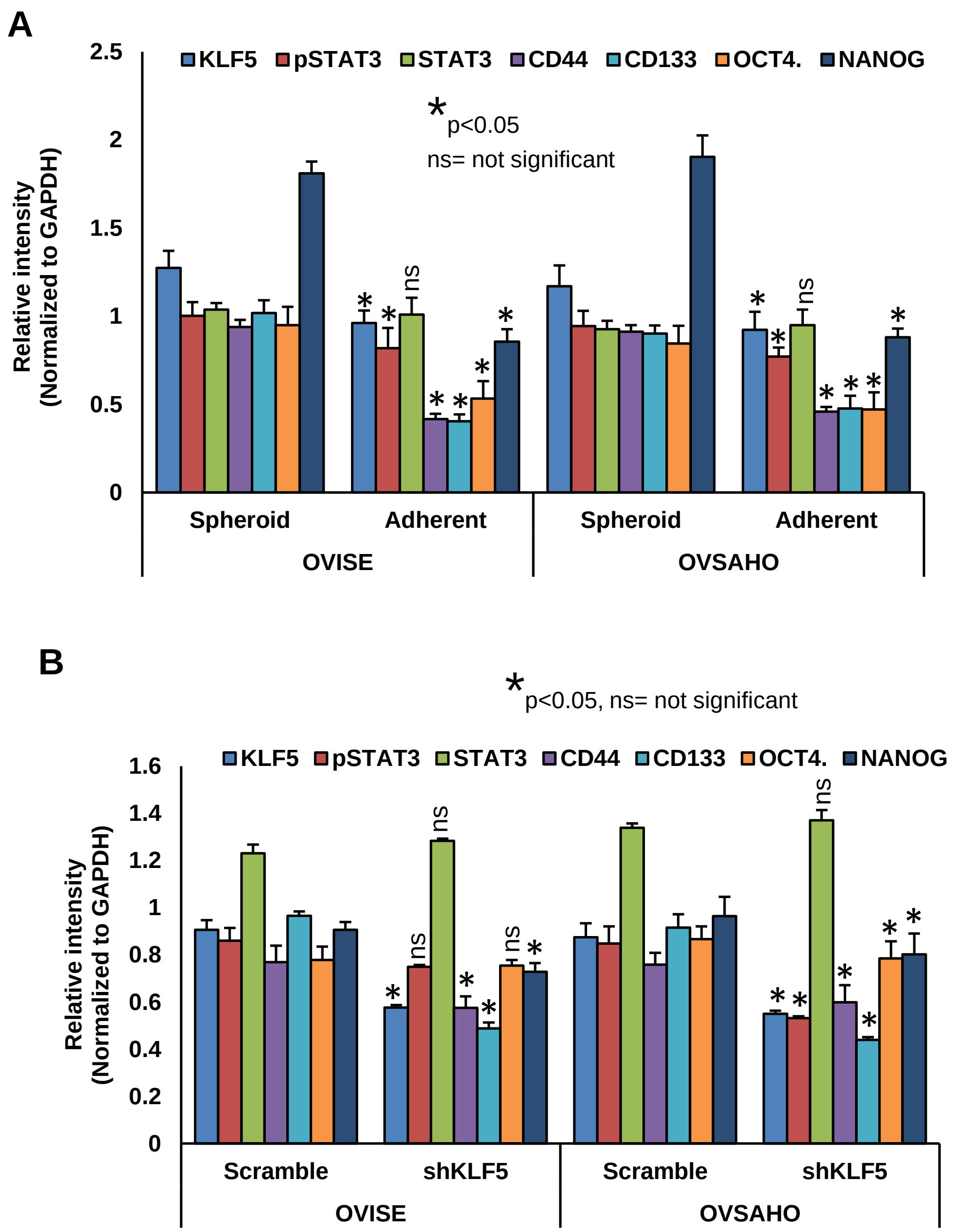

Supplementary Figure 7

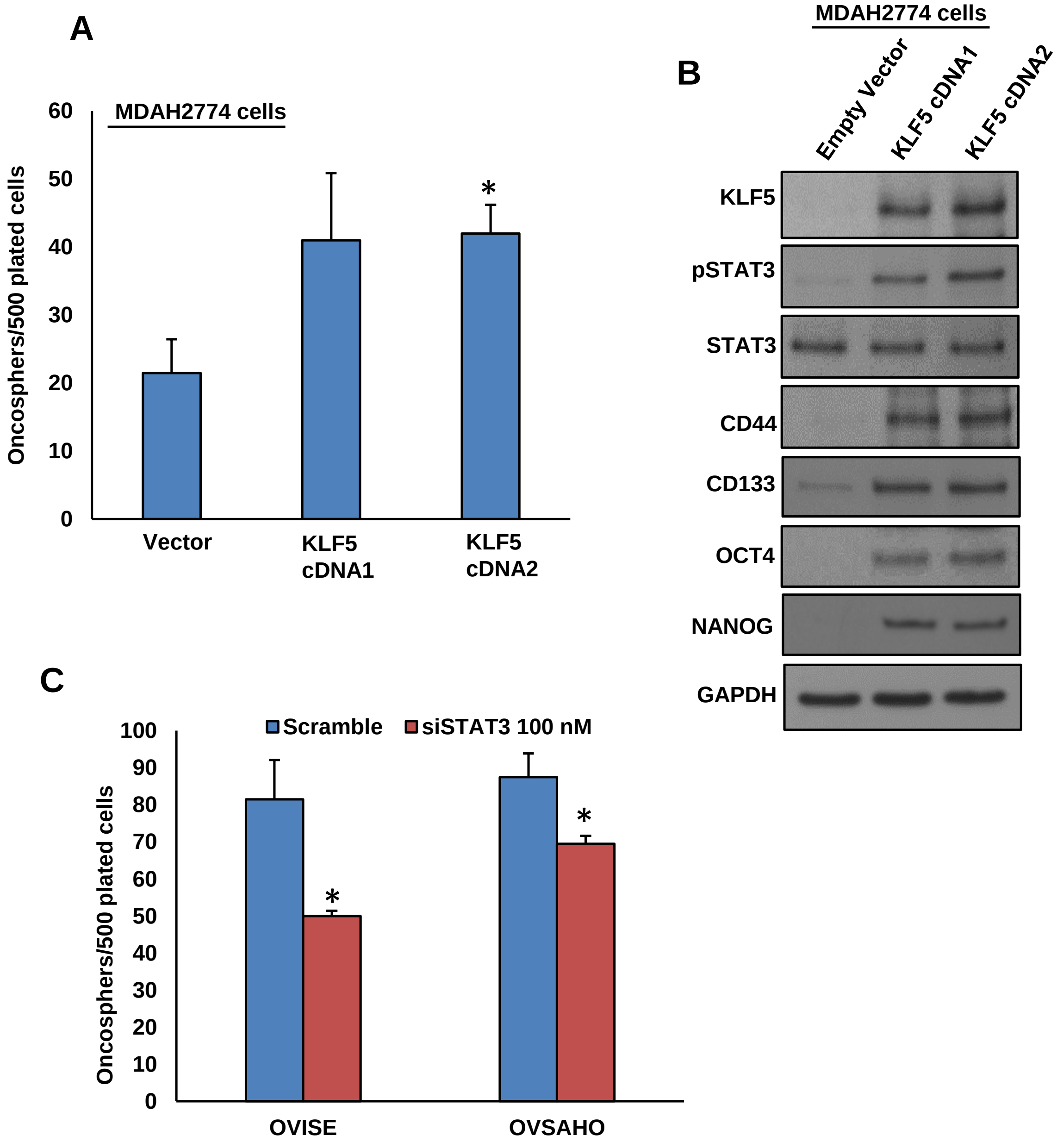

Supplementary Figure 8.

OVISE

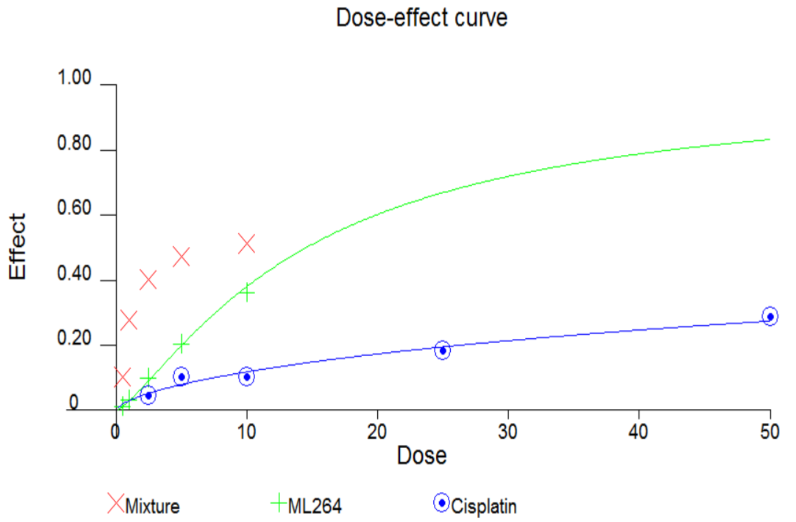

OVISE

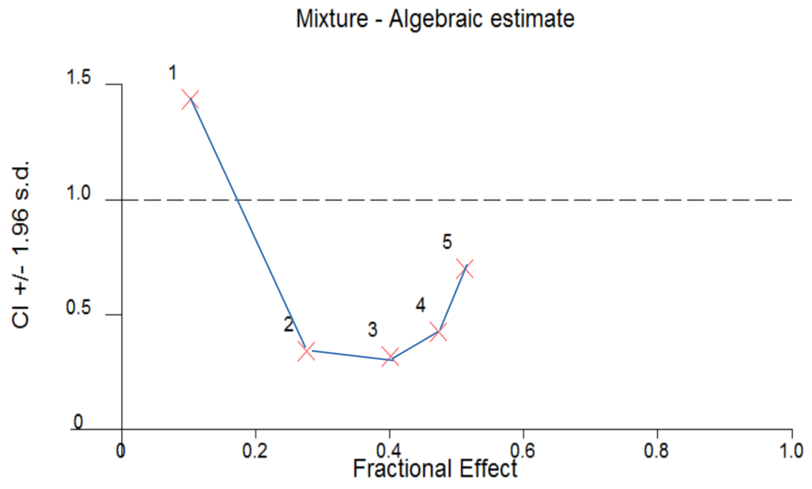

OVISE

Combination index (CI) for experimental values			
**ML264 (μM)**	**Cisplatin (μM)**	**Fa**	**CI**
0.5	10	0.104	1.439
1	10	0.271	0.341
**2.5**	**10**	**0.401**	**0.317**
5	10	0.473	0.426
10	10	0.513	0.702

Supplementary Figure 9.

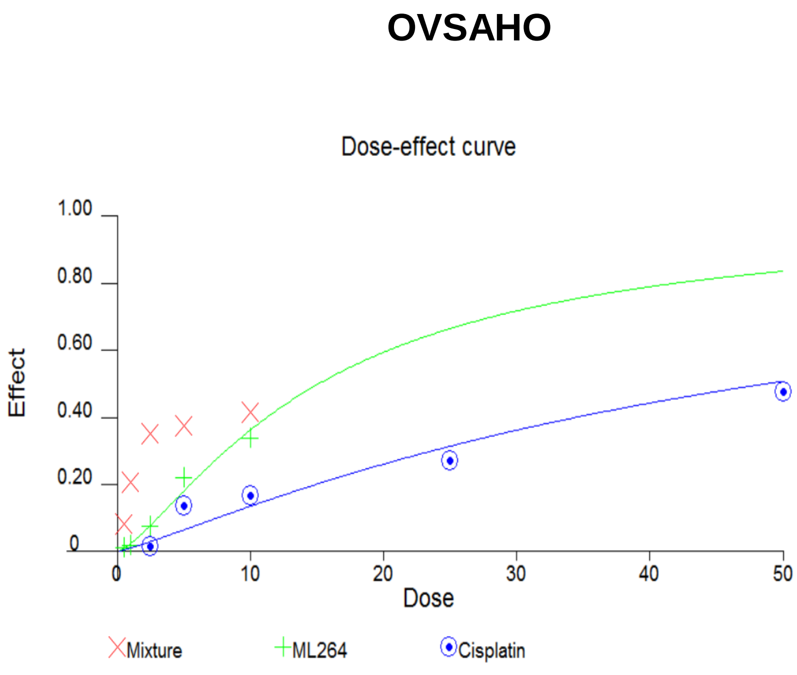

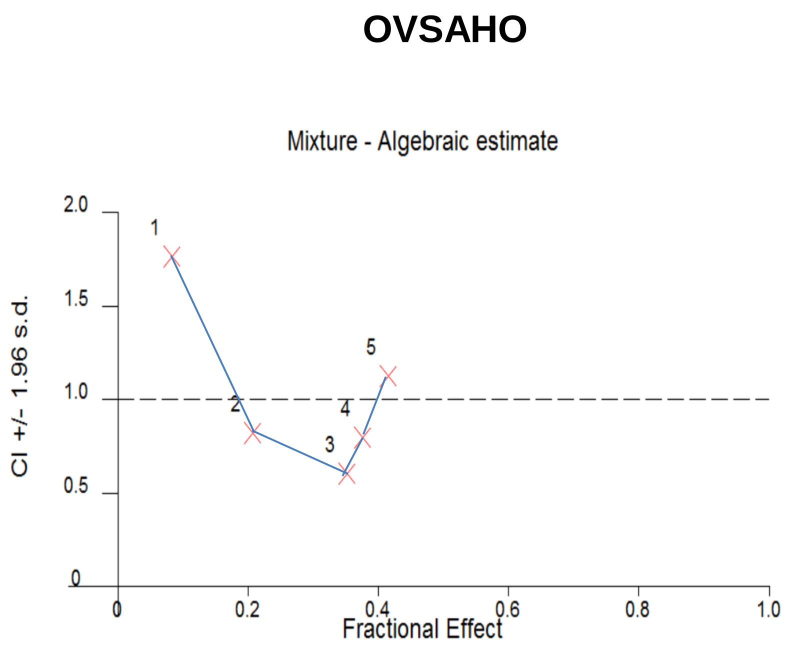

OVSAHO

Combination index (CI) for experimental values			
**ML264 (μM)**	**Cisplatin (μM)**	**Fa**	**CI**
0.5	10	0.084	1.769
1	10	0.207	0.824
**2.5**	**10**	**0.353**	**0.603**
5	10	0.376	0.796
10	10	0.415	1.126

Supplementary Figure 10

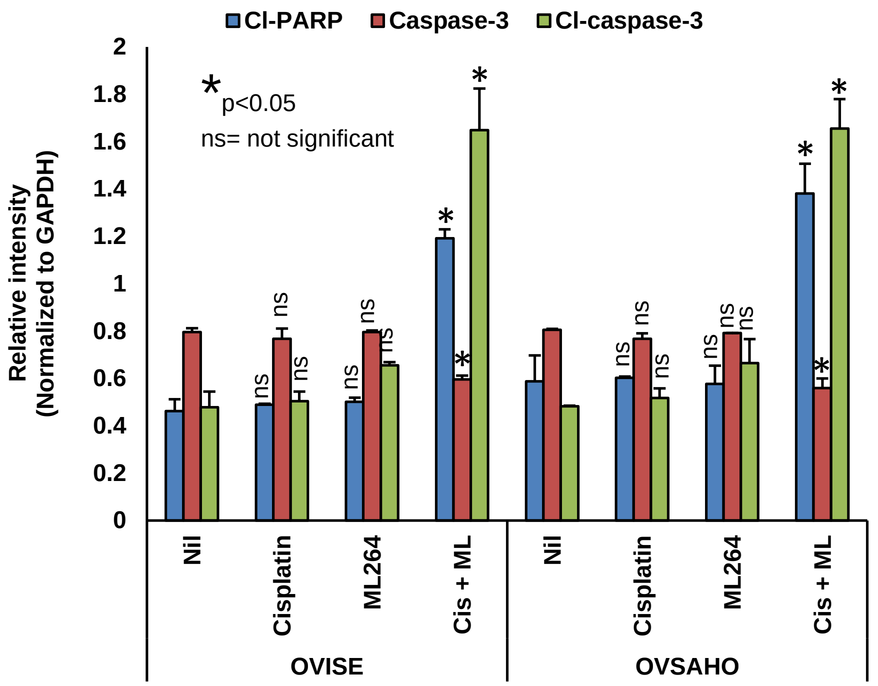

Supplementary Figure Legends

Supplementary Figure 1: (A) Basal expression of KLF5 and pSTAT3 in EOC cell lines. Proteins were isolated from six EOC cell lines and immunoblotted with antibodies against KLF5, pSTAT3, STAT3 and GAPDH (n=2). **(B)** Silencing of KLF5 inhibits STAT3 activation. EOC cells were transfected with scrambled siRNA and KLF5 siRNA (50 and 100 nM). After 48 hours, cells were lysed and proteins were immunoblotted with antibodies against KLF5, pSTAT3, STAT3 and GAPDH (n=2). **(C)** ML264 treatment down-regulates KLF5 expression and STAT3 activation in EOC cells. EOC cells were treated with indicated doses of ML264 for 48 hours. After cell lysis, equal amounts of proteins were separated by SDS-PAGE, transferred to immobilon membrane, and immuno-blotted with antibodies against KLF5, pSTAT3, STAT3 and GAPDH as indicated (n=2). **(D)** Knockdown of STAT3 has no effect on KLF5 expression. EOC cells were transfected with scrambled siRNA and STAT3 siRNA (50 and 100 nM). After 48 hours, cells were lysed and proteins were immunoblotted with antibodies against pSTAT3, STAT3, KLF5 and GAPDH (n=2). **(E)** Forced expression of KLF5 increases STAT3 activation. MDAH2774 cells were transfected with either empty vector or *KLF5* cDNA for 48 hours. Proteins were isolated and immunoblotted with antibodies against KLF5, pSTAT3, STAT3 and GAPDH for equal loading (n=2). *Indicates a statistically significant difference compared to respective control with $p < 0.05$.

Supplementary Figure 2. (A) ML264 treatment down-regulates the expression of EMT markers in EOC cells. EOC cells were treated with indicated doses of ML264 for 48 hours. After cell lysis, equal amounts of proteins were separated by SDS-PAGE, transferred to

immobilon membrane, and immuno-blotted with antibodies against KLF5, pSTAT3, STAT3, E-cadherin, N-cadherin, Vimentin, Twist, MMP-2, MMP-9 and GAPDH as indicated (n=2). **(B)** Silencing of KLF5 down-regulates the expression of EMT markers in EOC cells. EOC cells were transfected with scrambled siRNA and KLF5 siRNA (50 and 100 nM). After 48 hours, cells were lysed and proteins were immunoblotted with antibodies against KLF5, pSTAT3, STAT3, E-cadherin, N-cadherin, Vimentin, Twist, MMP-2, MMP-9 and GAPDH (n=2). *Indicates a statistically significant difference compared to respective control with $p < 0.05$.

Supplementary Figure 3. (A) Forced expression of KLF5 increased cell invasion. MDAH2774 cells were transfected with either empty vector or *KLF5* cDNA for 48 hours, cells were seeded into the upper compartment of invasion chambers. The bottom chambers were filled with RPMI media. After 24 h incubation, invaded cells were fixed, stained and quantified. **(B)** Forced expression of KLF5 increased cell migration. MDAH2774 cells were transfected with either empty vector or *KLF5* cDNA for 48 hours, cells were seeded into the upper compartment of migration chambers. The bottom chambers were filled with RPMI media. After 24 h incubation, migrated cells were fixed, stained and quantified. **(C)** Forced expression of KLF5 increased EMT progression. MDAH2774 cells were transfected with either empty vector or *KLF5* cDNA for 48 hours. Proteins were isolated and immunoblotted with antibodies against KLF5, pSTAT3, STAT3, E-cadherin, N-cadherin, Vimentin, TWIST, MMP-2, MMP-9 and GAPDH. Data

presented in the bar graphs are the mean ± SD of two independent experiments. *Indicates a statistically significant difference compared to control with $p < 0.05$.

Supplementary Figure 4. Effect of STAT3 Knockdown on invasion, migration and EMT. (A) Knockdown of STAT3 reduced cell invasion. EOC cells were transfected with scrambled siRNA and STAT3 siRNA (50 and 100 nM). After 48 hours, cells were seeded into the upper compartment of invasion chambers. The bottom chambers were filled with RPMI media. After 24 h incubation, invaded cells were fixed, stained and quantified. (B) Knockdown of STAT3 reduced cell migration. EOC cells were transfected with scrambled siRNA and STAT3 siRNA (50 and 100 nM). After 48 hours, cells were seeded into the upper compartment of migration chambers. The bottom chambers were filled with RPMI media. After 24 h incubation, migrated cells were fixed, stained and quantified. (C) Silencing of STAT3 down-regulates the expression of EMT markers in EOC cells. EOC cells were transfected with scrambled siRNA and STAT3 siRNA (50 and 100 nM). After 48 hours, cells were lysed and proteins were immunoblotted with antibodies against pSTAT3, STAT3, KLF5, E-cadherin, N-cadherin, Vimentin, Twist, MMP-2, MMP-9 and GAPDH. Data presented in the bar graphs are the mean ± SD of two independent experiments. *Indicates a statistically significant difference compared to control with $p < 0.05$.

Supplementary Figure 5. (**A**) ML264 treatment causes inactivation of AKT and Bad proteins in EOC cells. EOC cells were treated with 5 and 10 µM ML264 for 48 hours. Following treatment, cells were lysed and immunoblotted with antibodies against p-AKT, AKT, p-Bad and GAPDH (n=2). (**B)** ML264 treatment downregulates the expression of anti-apoptotic proteins and inhibitors of apoptosis in EOC cells. EOC cells were treated with indicated doses of ML264 for 48 hours. After cell lysis, equal amounts of proteins were separated by SDS-PAGE, transferred to immobilon membrane, and immuno-blotted with antibodies against Bcl-2, Bcl-xl, XIAP, CIAP1 and GAPDH as indicated (n=2). (**C**) Effect of ML264 on Bax activation in EOC cells. EOC cells were treated with ML264 for indicated time periods. Following treatment, cells were lysed in 1% Chaps lysis buffer and subjected to immuno-precipitation with anti-Bax 6A7 monoclonal antibody and probed with specific polyclonal anti-Bax antibody (top band) for detection of conformationally changed Bax protein. In addition, the total cell lysates (bottom band) were applied directly to SDS–PAGE, transferred to immobilon membrane and immuno-blotted with specific anti-Bax polyclonal antibody. (**D**) Activation of caspases and cleavage of PARP induced by ML264 treatment in EOC cells. EOC cells were treated with and without 5 and 10 µM ML264 for 48 hours. Cells were lysed, equal amount of proteins were separated on SDS-PAGE and immuno-blotted with antibodies against caspase-9, caspase-3, cleaved caspase-3, PARP and GAPDH (n=2). *Indicates a statistically significant difference compared to control with $p < 0.05$.

Supplementary Figure 6. (A) Isolation of spheroid-forming cells from EOC cells. Sphere forming assay was performed by culturing EOC cells (5 x 10^2 cells/well) in sphere medium for 14 days in 24-well ultra-low attachment plates. Proteins were isolated from spheroid-forming cells and respective parental adherent cells and immunoblotted with antibodies against KLF5, pSTAT3, STAT3, CD44, CD133, NANOG, OCT4 and GAPDH (n=2)**. (B)** Silencing of KLF5 inhibits stemness of spheroids as confirmed by immunoblotting using stem cell markers. EOC cells were transfected with scramble or KLF5 shRNA's and grown in sphere medium. Proteins were isolated from spheroids and immunoblotted with antibodies against KLF5, pSTAT3, STAT3, CD44, CD133, NANOG, OCT4 and GAPDH (n=2)**.** *Indicates a statistically significant difference compared to control with $p<0.05$.

Supplementary Figure 7. (A) Forced expression of KLF5 increased self-renewal ability of spheroids. MDAH2774 cells were transfected with either empty vector or *KLF5* cDNA and cells were subjected to sphere forming assay. Spheroids in the entire well were counted. **(B)** Forced expression of KLF5 increased stemness. MDAH2774cells were transfected with either empty vector or *KLF5* cDNA and grown in sphere medium. Proteins were isolated from spheroids and immunoblotted with antibodies against KLF5, pSTAT3, STAT3, CD44, CD133, NANOG, OCT4 and GAPDH. **(C)** Silencing of STAT3 decreased self-renewal ability of spheroids. EOC cells were transfected with scrambled siRNA and STAT3 siRNA (100 nM) and cells were subjected to sphere forming assay. Spheroids in the entire well were counted. Data presented in the bar graphs are the mean

± SD of two independent experiments. *Indicates a statistically significant difference compared to control with $p < 0.05$.

Supplementary Figure 8. Synergistic inhibition of cell viability by ML264 and cisplatin in OVISE cells. OVISE cells were treated with various combinations of ML264 and cisplatin for 48 h and dose effect (A) and Fractional effect (B) graphs were generated using Calcusyn software. Combination Index (CI) were calculated using Chou and Talalay method.

Supplementary Figure 9: Synergistic inhibition of cell viability by ML264 and cisplatin in OVSAHO cells. OVSAHO cells were treated with various combinations of ML264 and cisplatin for 48 h and dose effect (A) and Fractional effect (B) graphs were generated using Calcusyn software. Combination Index (CI) were calculated using Chou and Talalay method.

Supplementary Figure 10: ML264 potentiate cisplatin to induce the cleavage of caspase-3 and PARP. EOC cells were treated with indicated doses of ML264 and cisplatin either alone or in combination for 48 hours. Proteins were isolated and were immunoblotted with antibodies against caspase-3, cleaved caspase-3, PARP and GAPDH (n=2). *Indicates a statistically significant difference compared to control with $p<0.05$.
